# Heme Oxygenase-1 Inhibition Potentiates the Effects of Nab-Paclitaxel-Gemcitabine and Modulates the Tumor Microenvironment in Pancreatic Ductal Adenocarcinoma

**DOI:** 10.3390/cancers13092264

**Published:** 2021-05-08

**Authors:** Iman M. Ahmad, Alicia J. Dafferner, Kelly A. O’Connell, Kamiya Mehla, Bradley E. Britigan, Michael A. Hollingsworth, Maher Y. Abdalla

**Affiliations:** 1 College of Allied Health Professions, University of Nebraska Medical Center, Omaha, NE 68198, USA; iman.ahmad@unmc.edu; 2 Department of Pathology and Microbiology, University of Nebraska Medical Center, Omaha, NE 68198, USA; adafferner@unmc.edu; 3 Fred & Pamela Buffett Cancer Center, The Eppley Institute for Research in Cancer, University of Nebraska Medical Center, Omaha, NE 68198, USA; koconnell@unmc.edu (K.A.O.); kamiya.mehla@unmc.edu (K.M.); mahollin@unmc.edu (M.A.H.); 4 Veterans Affairs Medical Center-Nebraska Western Iowa, Department of Internal Medicine and Research Service, Omaha, NE 68105, USA; bradley.britigan@unmc.edu

**Keywords:** heme oxygenase-1, PDAC, chemotherapy, immune cells

## Abstract

**Simple Summary:**

Pancreatic ductal adenocarcinoma (PDAC) is one of the most aggressive malignancies. Chemotherapy has had limited success in extending the life span of patients with advanced PDAC. Thus, new treatment approaches are needed. Heme oxygenase-1 (HO-1) is a key enzyme for the protection of cells against oxidative stress. Interestingly, HO-1 is overexpressed in several cancers, including PDAC. In the present study, our findings demonstrate the novel role of HO-1 in vivo in PDAC progression and in sensitizing tumor cells to cytotoxic chemotherapy by modulating the tumor microenvironment (TME).

**Abstract:**

Pancreatic ductal adenocarcinoma (PDAC) is an aggressive malignancy with a poor prognosis. Tumor hypoxia plays an active role in promoting tumor progression, malignancy, and resistance to therapy in PDAC. We present evidence that nab-paclitaxel–gemcitabine (NPG) and/or a hypoxic tumor microenvironment (TME) up-regulate heme oxygenase-1 (HO-1), providing a survival advantage for tumors. Using PDAC cells in vitro and a PDAC mouse model, we found that NPG chemotherapy up-regulated expression of HO-1 in PDAC cells and increased its nuclear translocation. Inhibition of HO-1 with ZnPP and SnPP sensitized PDAC cells to NPG-induced cytotoxicity (*p* < 0.05) and increased apoptosis (*p* < 0.05). Additionally, HO-1 expression was increased in gemcitabine-resistant PDAC cells (*p* < 0.05), and HO-1 inhibition increased GEM-resistant PDAC sensitivity to NPG (*p* < 0.05). NPG combined with HO-1 inhibitor inhibited tumor size in an orthotopic model. In parallel, HO-1 inhibition abrogated the influx of macrophages and FoxP3+ cells, while increasing the proportion of CD8+ infiltration in the pancreatic tumors. These effects were mediated primarily by reducing expression of the immunosuppressive cytokine IL-10.

## 1. Introduction

Pancreatic ductal adenocarcinoma (PDAC) is among the most common and lethal cancers, with more than 60,430 new cases annually [1] and a 5-year overall survival of around 10% [1]. PDAC is projected to be the second leading cause of cancer-related death in the United States by 2030 [2]. Although surgery is the standard of care for resectable PDAC, less than 20% of patients with pancreatic cancer are surgically treated at the time of diagnosis [3,4]. Conventional chemotherapy and radiotherapy are commonly employed as first-line treatment in patients with non-resectable or borderline-resectable tumors [5].

Hypoxia is a prominent feature of the tumor microenvironment in multiple solid tumors [6,7]. Hypoxia plays an active role in promoting tumor growth, progression, metastasis, and resistance to chemotherapy and radiation therapy [8,9]. In PDAC, HIF-1α is overexpressed in pancreatic tumor cells and is associated with cell proliferation [10,11]. One critical gene modulated by hypoxia is heme oxygenase-1 (HO-1). Our lab and others have reported that hypoxia induces HO-1 expression in pancreatic cancer cells [12,13,14]. Additionally, multiple chemotherapeutic agents, including nab-paclitaxel, induce HO-1 in treated cancer cells [15].

HO-1 is a cytoprotective enzyme that catalyzes the degradation of heme into ferrous iron (Fe), biliverdin, and carbon monoxide (CO) [16]. Biliverdin and bilirubin have antioxidant properties and are considered endogenous reactive oxygen species (ROS) scavengers [17]. CO has antiapoptotic and anti-inflammatory effects and was shown to play a role in many inflammation-related diseases [18,19,20].

To date, there are three known isoforms of HO: HO-1, HO-2, and HO-3. The constitutively expressed isoform is HO-2, whereas HO-1 is the inducible form. Hypoxia, cancers, infections, metals, and oxidative stress are known to induce HO-1 [21,22,23].

Nab-paclitaxel plus gemcitabine is a first-line treatment for metastatic PDAC [24]. The combination of gemcitabine and the albumin-bound paclitaxel (nab-paclitaxel, Abraxane) provides a 2-month survival benefit in patients with advanced PDAC [25]. The synergistic effect of the combination was attributed to an improvement in tumor delivery for both gemcitabine and paclitaxel [24]. Despite effectively minimizing tumor progression, the results achieved by current treatment regimens are accompanied by considerable side effects and fail in significantly improving patient survival [5,26]. Therefore, novel therapeutic combinations are desperately needed to treat PDAC.

The present study investigated the role of HO-1 in chemoresistance and in provoking an immunosuppressive TME in PDAC. Our results show that NPG treatments up-regulate HO-1 in pancreatic cancer cell lines. Inhibition of HO-1 sensitizes PDAC and chemotherapy-resistant PDAC cells to NPG therapy by increasing ROS, disrupting GSH, and increasing apoptosis. Finally, we demonstrated that HO-1 inhibitors abrogate an immunosuppressive TME. Inhibition of HO-1 could be a strategy to enhance the response of PDAC to chemotherapeutic drugs.

## 2. Materials and Methods

### 2.1. Cell Culture and Experimental Reagents

The human pancreatic cancer cell lines Capan-1, CD18/HPAF, Mia Paca-2, and T3M4 were obtained from American Type Culture Collection and cultured in DMEM with 7% heat-inactivated fetal bovine serum. Cells were carried out in a 5% CO_2_ incubator at 37 °C. Gemcitabine HCl was obtained from LC Laboratories (Woburn, MA, USA). Abraxane was a kind gift from Celgene (Summit, NJ, USA). Tin protoporphyrin IX dicholoride, zinc protoporphyrin, and ferrostatin-1 were obtained from Santa Cruz Biotechnology (Dallas, TX, USA). Erastin (a positive control for Ferroptosis) was purchased from Cayman Chemical (Ann Arbor MI).

The gemcitabine-resistant pancreatic cancer cell lines (T3M4, and MiaPaca-2) were a kind gift from the laboratory of Dr. Pankaj Singh, as described before [27].

### 2.2. Preparation of Cytosolic and Nuclear Extracts

The Abcam Standard Cell Fractionation Kit (AB109719) was used according to the manufacturer’s protocol to obtain nuclear, mitochondrial, and cystosolic fractions from cell lines plated in 6-well plates. Two wells of a six-well plate were pooled together to obtain 2.4 × 106 cells for each sample. Briefly, Buffer B was added, and the samples were centrifuged to extract the cytosolic fraction. Buffer C was added to the remaining sample and centrifuged to obtain the mitochondrial fraction. The remaining sample was resuspended in Buffer A to obtain the nuclear fraction. Four volumes of sample were mixed with one volume of loading buffer, denatured for 10 min at 60 °C, and then immediately loaded onto 12% SDS polyacrylamide gel for Western blot analysis.

### 2.3. Western Blots

Protein was collected from cell cultures using RIPA Lysis and Extraction Buffer (Thermo Fisher Scientific, Waltham, MA, USA) with EDTA and protease inhibitors. Protein in the cell lysates was quantified using the DC Protein Assay (Bio-Rad, Hercules, CA, USA), and 50 µg/well were run on a 10% sodium dodecyl sulfate (SDS) polyacrylamide gel in Tris/Glycine/SDS buffer. Proteins were transferred to polyvinylidene difluoride (PVDF) membranes activated with 100% methanol in Tris/Glycine buffer. The blots were blocked with 5% nonfat milk in Tris-buffered saline with Tween 20 (TBST), and hybridized overnight at 4 °C with primary antibodies against HO-1 (Enzo, Farmingdale, NY, USA), SOD-1 (Abcam, Cambridge, MA, USA), and beta actin (Cell Signaling Technologies, MA, USA). Blots were washed with TBST, incubated with horseradish peroxidase tagged secondary antibody (Cell Signaling Technologies) at room temperature, and washed again with TBST. The membranes were treated with Radiance Plus Femtogram HRP substrate solution (Azure Biosystems, Dublin, CA, USA), and the proteins of interest were imaged using an Azure c600 Imaging System.

### 2.4. Apoptosis Assay

Apoptosis was analyzed via Flow Cytometry using the Annexin V_FITC Apoptosis Detection Kit (eBioscience, San Diego, CA, USA). Cells were detached from culture plates with accutase and washed with phosphate-buffered saline (PBS). Cells were incubated with annexin V-FITC and propidium iodide in binding buffer. Cells were analyzed by the UNMC flow cytometry core facility on a FACSCalibur apparatus (BD, Franklin Lakes, NJ, USA). Data were analyzed with FlowJo software v7.0 (TreeStar, Inc., Ashland, OR, USA), to identify live cells, necrosis, early apoptosis, and late apoptosis.

### 2.5. CellTiter 96 Non-Radioactive Cell Proliferation Assay (MTT)

Cell lines were plated out into 96-well tissue culture plates and incubated for 24 h at 37 °C with 5% CO_2_. Cells were then treated with different reagents and incubated for 24 h at 37 °C. MTT assay was performed using Promega’s MTT assay kit (#G4000) according to the manufacturer’s protocol. Plates were read on a BioTek Synergy H1 plate reader at 570 nm.

### 2.6. Confocal Microscopy

Cells were cultured in 4-well culture slides (BD Biosciences, Franklin Lakes, NJ, USA), washed once with phosphate buffered saline (PBS), and then treated for overnight at 37 °C. Cells were then treated with 0.1% Triton X-100 to permeabilize the cells and blocked in 1% goat serum. HO-1 antibody was incubated overnight at 4 °C. The secondary antibody goat anti-rabbit IgG (H + L) (Invitrogen #A11034, Alexa Flour 488) was added for 1 h and mounted with DAPI-containing mounting media (Vectashield #H-1500). For ROS experiments, dihydroethidium (DHE), and dichloro-dihydro-fluorescein diacetate (DCFH-DA) reagents were added for 30 min at 37 °C. Slides were analyzed using Zeiss LSM 710.

### 2.7. Glutathione (GSH) Analysis

Cell cultures were washed with cold phosphate-buffered saline (PBS) and detached using a cell scraper. The cells were centrifuged at 4 °C for 10 min at 200× *g*, then resuspended in 10 mM hydrochloric acid, and vortexed. The cells were lysed by 2 freeze/thaw cycles. Protein was quantified using DC Protein Assay (Bio-Rad, Hercules, CA, USA). GSH, and GSSG were measured using a GSH Quantification kit (DOJINDO Inc., Rockville, MD, USA) according to the manufacturer’s guidelines.

### 2.8. In Vivo Mice Model Studies

The animal experiments were approved by the Institutional Animal Care and Use Committee (IACUC) at the University of Nebraska Medical Center (UNMC). Female 6-week-old C57BL/6 mice were obtained from The Jackson Laboratory (Bar Harbor, ME, USA) and maintained in pathogen-free conditions in the UNMC animal facility. Luciferase-tagged KPC cells were orthotopically implanted into each pancreas (4000 cells/mouse). Seven days post implantation, the mice were randomly placed into 4 groups with 5–6 mice per group and imaged by in vivo imaging system (IVIS) to ensure all mice had a tumor. Group 1 was a control with vehicle treatment, group 2 was given 5 mg/kg SnPP 3×/week, group 3 was given 60 mg/kg gemcitabine and 5 mg/kg Abraxane 2×/week, and group 4 was given 60 mg/kg gemcitabine and 5 mg/kg Abraxane 2×/week along with 5 mg/kg SnPP 3×/week. Treatment was started on day 8 and continued through day 29. On day 30 all of the animals were sacrificed by CO_2_ asphyxiation followed by cervical dislocation. The tumors were removed, measured, and weighed. Tumor volume (mm^3^) measured with calipers and calculated as (W2 × L) / 2, where W is width and L is length. The primary tumors and any remaining pancreatic tissue were collected for RNA isolation, immunohistochemistry (IHC), and protein analysis.

### 2.9. Immunohistochemistry (IHC) and Confocal Studies

Primary pancreatic tumors and the remaining pancreatic tissue were fixed in 10% buffered formalin for 3 days, rinsed in 70% ethanol overnight, embedded in paraffin, and then 4 µM thick sections were sectioned into unstained slides. Slides were deparaffinized with xylene, rehydrated through decreasing alcohol gradients, and washed in PBS; then, endogenous peroxidases were quenched in 3% hydrogen peroxide. Antigen retrieval was performed with sodium citrate buffer (pH 6) in a microwave for 15 min. Sections were blocked using 2.5% normal horse serum for 1 h and incubated with primary CC3 antibody (Cell Signaling Technologies #9664S) at 1:100 dilution, FOXP3 antibody (Invitrogen #14-4777-80) at 1:100 dilution, F4/80 antibody (GeneTex #GTX26640) at 1:100 dilution, CD4 antibody (Abcam #AB13685) at 1:1500 dilution, and/or CD8 antibody (Abcam #AB209775) at 1:1500 dilution overnight at 4 °C. Sections were incubated with ImmPRESS universal polymer reagent (Vector Laboratories, San Fransisco, CA, USA) at room temperature for 1 h. The slides were washed well with PBS, stained using 3,3′-diaminobenzidine (DAB) substrate kit (Vector Laboratories, San Fransisco, CA, USA) for 2 min, and counterstained with hematoxylin. The stained slides were dehydrated, washed with xylene, and mounted. For confocal studies, after washing with PBS, slides were incubated with anti-rabbit FITC-conjugated IgG antibodies for 1 h at room temperature. Slides were then washed, mounted, and imaged using a Zeiss LSM710 confocal microscope at UNMC core facility.

### 2.10. RNA

Total RNA extraction was performed using RNeasy Plus Mini Kit (Qiagen), according to the manufacturer’s instructions. RNA integrity and yield were determined using a NanoDrop 1000.

### 2.11. Eukaryotic RNA Seq

Sequencing libraries were generated by the UNMC NGS Core beginning with 200 ng of total RNA from each sample using the TrueSeq Stranded Total RNA library kit from Illumina, following the recommended procedures (Illumina Inc., San Diego, CA, USA). Resultant libraries were assessed for size of insert by analysis of an aliquot of each library on a Bioanalyzer instrument (Agilent Technologies, Santa Clara, CA, USA). Each library possesses a unique indexing identifier barcode allowing the individual libraries to be multiplexed together. Multiplexed libraries (12 samples per pool) were sequenced on two 150-cycle mid output flow cells of the NextSeq500 DNA Analyzer (Illumina) to generate a total of approximately 20 to 25 million 75 bp read pairs for each sample. FASTQ files were provided to the UNMC Bioinformatics Core Facility for further analysis.

Data analysis was performed with at the Bioinformatics Core at UNMC. Genes with a cutoff value log fold change (±2) were selected, and those with zero counts in either control or test samples were excluded for stringency. The Ingenuity Pathway Knowledge Base (IPA) was used to identify the enriched cellular and molecular functions among the differentially expressed transcripts in all samples.

### 2.12. TCGA Bioinformatics Analysis

Molecular expression by RNAseq in a PDAC patients was obtained from The Cancer Genome Atlas (TCGA) UCSC Xena website (https://xenabrowser.net/, accessed December 2020) as previously described [28].

### 2.13. Statistical Analysis

All data are representative of at least 3 independent experiments and presented as the mean ±SEM. Statistical significance was determined by 1-way/2-way ANOVA, or student’s t test. A P value of less than 0.05 was considered as statistically significant. Analyses were performed with GraphPad Prism version 5.0 for Windows (GraphPad Software, San Diego, CA, USA).

## 3. Results

### 3.1. HO-1 Expression in Human Pancreatic Tissues Correlates with Clinical Data

**Figure 1:**
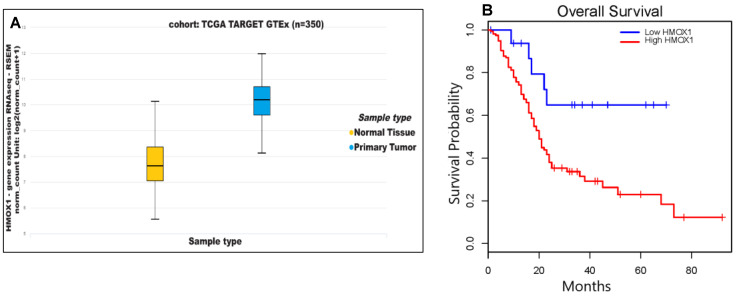
HO-1 expression in human pancreatic tissues correlates with clinical data. (**A**) Expressions of mRNA levels of HMOX1 in normal tissues (*n* = 167) and primary PDAC tumors (*n* = 178). (**B**) Correlation of HMOX1 expression and overall survival in PDAC patients with high HO-1 expression (*n* = 160) as compared to low HO-1 expression (*n* = 18) using Kaplan–Meier analysis.

We analyzed HO-1 gene (HMOX1) expression data obtained from The Cancer Genome Atlas (TCGA) and found significantly higher pancreatic HMOX1 expression in PDAC tumors as compared to normal tissues (*p* < 0.05) (Figure 1A). Kaplan–Meier analysis of survival probability for PDAC patients revealed that patients with lower HMOX1 expression showed longer survival probability than patients with higher HMOX1 (*p* = 0.013) (Figure 1B). These TCGA clinical data are consistent with our previous findings [12], and led us to posit that higher expression of HO-1 contributes to PDAC lethality, and that lowering HO-1 expression may improve prognosis in PDAC patients.

### 3.2. NPG Induces Ho-1 Expression in PDAC Cells through P38 Pathway and Increases Nuclear Translocation of HO-1

**Figure 2:**
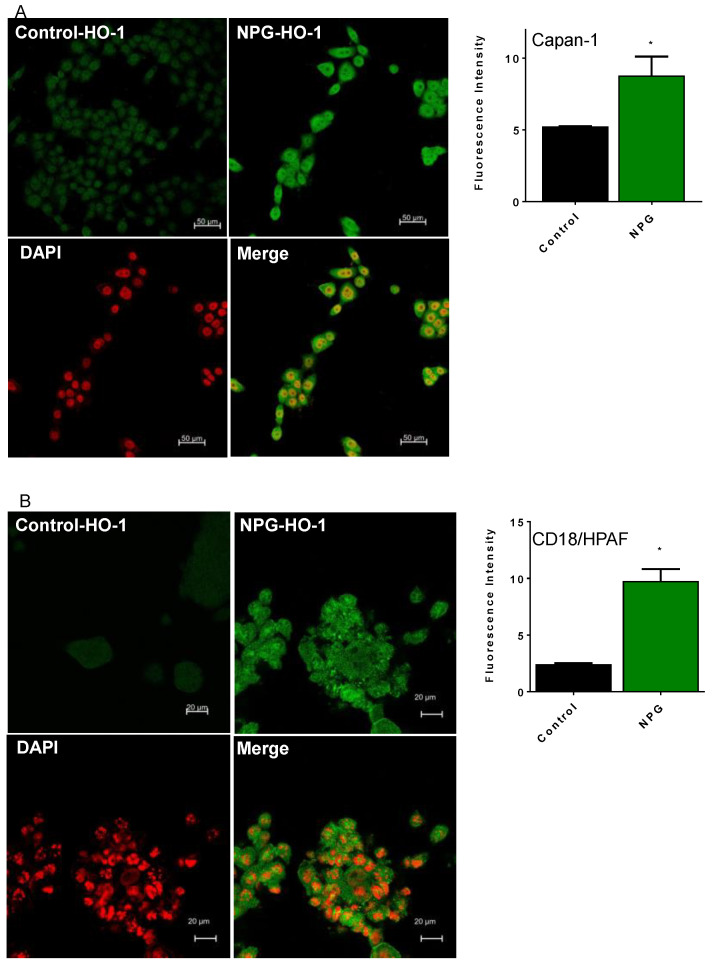

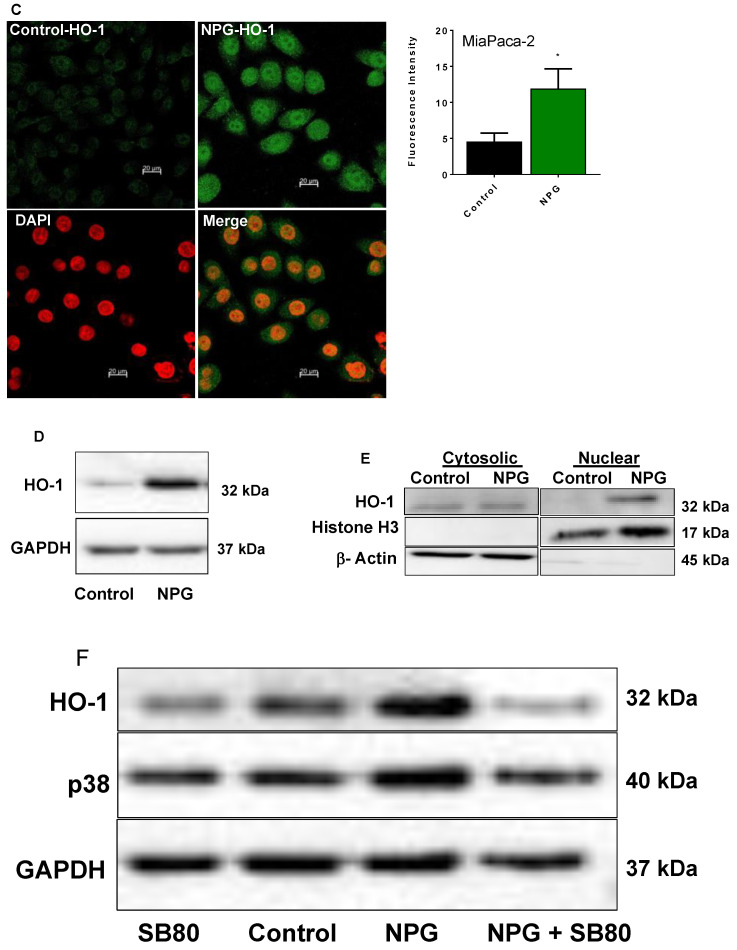
NPG increases HO-1 expression and induces nuclear enrichment in PDAC cells. PDAC cells were treated with NPG for 24 h and stained with anti-HO-1 antibody. Counterstaining of cells was performed by using the nuclear dye DAPI (red), with study by confocal microscopy. NPG treatment induces HO-1 expression in PDAC cell lines Capan-1 (**A**), CD18/HPAF (**B**), and MiaPaca-2 (**C**). Fluorescence intensity of HO-1 is shown on the right side of each panel. (**D**) NPG increases HO-1 in T3M4 cells (immunoblotting). (**E**) NPG induces HO-1 translocation to the nucleus (analysis of cellular fractionation and subcellular localization of HO-1 in MiaPaca-2 cells). The densitometric analysis of fluorescence intensity for HO-1 is shown on the right side of each cell line. (**F**) p38 inhibitor (SB203580) reduced HO-1 induction in Capan-1 cells (shown are representative figures, *n* = 3, * *p* < 0.05). Please find the western bolt in supplementary file 1.

We treated different PDAC cells with NPG for 24 h and evaluated HO-1 protein expression by confocal microscopy and Western blots. As shown in Figure 2, treatment with NPG induced higher levels of HO-1 in Capan-1 (A), CD18/HPAF (B), and MiaPaca-2 (C) cells as determined by increased fluorescence (Figure 2A–C). Western blots of PDAC cells showed similar results, where NPG increased HO-1 protein expression (Figure 2D,E). Interestingly, NPG treatment induced nuclear localization of HO-1, as shown by confocal images and cellular fractionation (Figure 2A–C).

HO-1 expression is known to be regulated by the mitogen-activated protein kinase (MAPK)-p38 signaling system [21,29,30]. Therefore, we examined NPG effects on the expression of HO-1 via the p38 signaling pathway. As shown in Figure 2F, NPG induced-HO-1 expression in PDAC cells is mediated through p38 pathway, as pretreatment of 10 μM of SB203580 (p38 inhibitor) reduced HO-1 expression in PDAC cells (Figure 2F).

### 3.3. Inhibition of HO-1 Reduces Proliferation and Enhances the Cytotoxic Effects of NPG in PDAC and GEM-Resistant PDAC Cells but Not Ferroptosis

**Figure 3:**
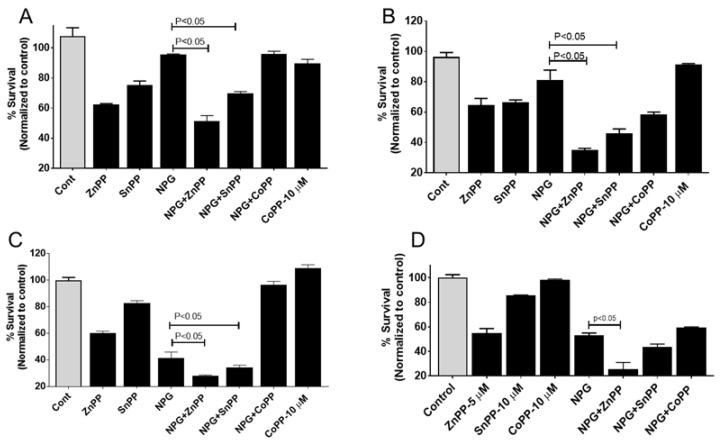

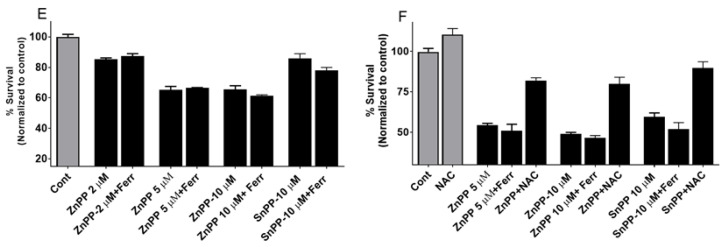
Inhibition of HO-1 sensitizes PDAC cells to NPG. The anti-proliferative effects of combined treatment with HO-1 inhibitors and NPG. PDAC cells were treated with NPG (5 µM GEM, and 0.1 µM nab-paclitaxel), or co-treated with 5 µM ZnPP, 10 µM SnPP, or 10 µM CoPP for 48 h, and viability was determined using the MTT assay. Vertical bars indicate the means ± SEM of 3 independent experiments. (**A**) MiaPaca-2, (**B**) Capan-1, and (**C**) S2-013 PDAC cell lines. *p* < 0.05 compared with the control or as indicated in the figure. (**D**) shows the inhibitory effects of HO-1 inhibition on mouse derived pancreatic cancer cell line, KPC. (**E**,**F**) show the effects of N-acetylcysteine (NAC) and ferrostatin-1 (Ferr) on HO-1 inhibitor-induced cell death. MiaPaca-2 (**D**) and PANC-1 (**E**) cells were treated with 5 μM ZnPP, or 10 μM SnPP in the presence or absence of 1 μM Ferr, or 10 mM NAC (PANC-1 cells only). The cells were treated with NAC, or Ferr for 2 h prior to HO-1 inhibitor treatment. Cell viability was assessed by MTT assay. Values are expressed as the means ± standard deviation of 3 measurements.

Previously, we showed that hypoxia induced HO-1 in PDAC cells, and that inhibiting HO-1 enhanced the cytotoxic effect of gemcitabine (GEM) [12]. As NPG induced HO-1 expression, we investigated the impact of HO-1 inhibitors on cell proliferation in NPG-treated PDAC cell lines. PDAC cells were treated with NPG for 24 h in the presence or absence of different HO-1 inhibitors. The results revealed that HO-1 inhibition significantly enhanced the effect of NPG in different PDAC cells (*p* < 0.05) (Figure 3). The addition of NPG (gemcitabine at 5 M, nab-paclitaxel at 0.1 M) to MiaPaca-2 cells modestly reduced cell survival to 95%, which was further reduced to 51% and 70% by the addition of HO-1 inhibitors ZnPP, and SnPP, respectively (Figure 3A, *p* < 0.05). Similarly, Capan-1 cell survival was significantly reduced to 35% by ZnPP and 46% by SnPP when combined with NPG as compared to NPG treatment alone (~80%) (Figure 3B, *p* < 0.05). Moreover, addition of ZnPP or SnPP in combination with NPG significantly reduced S2-013 cell survival to 28% or 34%, respectively, as compared to NPG treatment alone (~40%) (Figure 3C, *p* < 0.05)). Interestingly, similar effects of NPG and HO-1 inhibition were seen using the mouse-derived pancreatic cancer cell line, KPC (Figure 3D). KPC cell survival was reduced to 55% by ZnPP, and to 25% when combined with NPG (*p* < 0.05).

To confirm the cytoprotective roles of HO-1, CoPP (an HO-1 inducer) was added in combination with NPG. The addition of CoPP restored cell survival in MiaPaca-2, Capan-1, and S2-013 to 96%, 58%, and 96%, respectively (Figure 3A–C). This result indicates that HO-1 induction enhanced survival and proliferation of PDAC cells, and combining an HO-1 inhibitor with NPG increased the toxic effects of NPG. The addition of CoPP to cells either increased or had no effect on PDAC cells (Figure 3A–C).

Ferroptosis is a type of cell death that is biochemically different from other types of cell death [31]. Ferroptosis is characterized by ROS accumulation, lipid peroxidation, and GSH depletion [31,32]. Ferroptosis is associated with a variety of pathological processes and cancers, including pancreatic cancer [33]. To investigate if HO-1 inhibition would induce ferroptosis, we cultured our cells with HO-1 inhibitors (ZnPP or SnPP) in the presence of the ferroptosis inhibitor ferrostatin-1 (Ferr). Ferr could not reverse HO-1 inhibition-induced MiaPaca-2 cells death, as shown in Figure 3D. However, treatment with the ROS scavenger NAC (10 mM) abrogated the reduction in PANC-1 cell survival induced by treatment with HO-1 inhibitors, indicating a role for increased ROS in causing cell death during HO-1 inhibition (Figure 3F).

**Figure 4:**
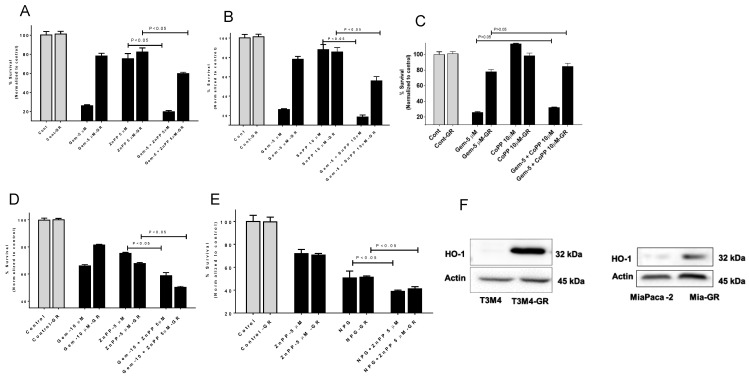
The cytotoxic effects of HO-1 inhibitors and GEM or NPG combination on GEM-resistant cells by MTT. GEM-resistant and parental PDAC cells were treated with GEM or NPG for 48 h and tested for proliferation. T3M4 and T3M4-GR were treated with GEM or GEM with HO-1 inhibitors ZnPP (**A**) or SnPP (**B**), or HO-1 inducer CoPP (**C**). (**D**) MiaPaca-2 or MiaPaca-2-GR show similar results. (**E**) T3M4–GR cells were more sensitive to NPG in combination with HO-1 inhibitor (ZnPP). (**F**) Western blot analysis shows that GR cells increased HO-1 expression as compared to parental cells. Data are presented mean ± SEM. Please find the western bolt in supplementary file 1.

To evaluate the effects of HO-1 inhibition on chemotherapy-resistant pancreatic tumor cell lines, we treated GEM-resistant (GR) cell lines MiaPaCa-2 and T3M4 and their parental cells with GEM or with NPG in the presence of the HO-1 inhibitors ZnPP and SnPP. Our findings collectively suggest that combining inhibitors of HO-1 with GEM or NPG therapy provides more potent antiproliferative and antitumor activity than either treatment alone (Figure 4A,B,D,E) (*p* < 0.05). This result indicates that HO-1 induction enhanced resistance of PDAC cells to the anticancer agents GEM alone or in combination with nab-paclitaxel (NPG). On the other hand, treating cells with the HO-1 inducer CoPP increased cell survival alone or in combination with GEM (Figure 4C). Interestingly, when PDAC and PDAC-GR cells were tested for HO-1 expression, a prominent increase in HO-1 was shown in GEM-resistant cells as compared to parent cells (Figure 4F).

### 3.4. NPG and HO-1 Inhibition Generates Reactive Oxygen Species (ROS) and Increases Oxidized Glutathione

**Figure 5:**
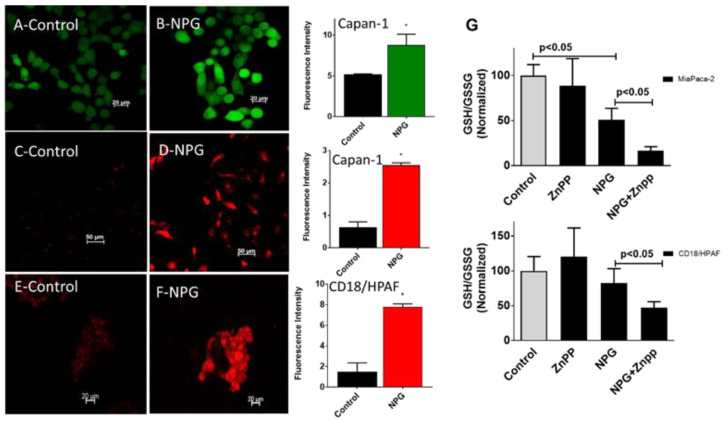
NPG treatment increases ROS production in PDAC cells. PDAC cells were treated with NPG (GEM 0.5 M, nab-paclitaxel 0.1 M) for 24 h. Subsequently, cells were preloaded with 2′,7′-dichlorodihydrofluorescein diacetate (DCF) or dihydroethidium (DHE) and studied under a fluorescent microscope. (**A**,**C**) show Capan-1 controls, and (**E**) shows CD18/HPAF cells control cells treated with vehicle only. (**B**) shows NPG-treated cells loaded with DCF, whereas (**D**,**F**) show NPG-treated cells loaded with DHE. The panel on the right side shows fluorescence intensity of the control and NPG-treated cells. (*p* < 0.05, *n* = 3). (**G**) NPG in combination with HO-1 inhibition significantly increased oxidized glutathione in both the MiaPaca-2 and CD18/HPAF cell lines. Data are presented mean ± SEM. * *p* < 0.05.

To investigate the effect of NPG on the oxidative status of PDAC cells, we treated cells with NPG for 24 h and tested for ROS production using the oxygen-sensitive probe dichlorodihydrofluorescein diacetate (DCFH-DA). For detecting intracellular superoxide (O2 •−), we used the O2 •− sensitive probe dihydroethidium (DHE). Treatment with NPG increased ROS production as detected by increasing DCF and DHE fluorescence in Capan-1 and CD18/HPAF cells (Figure 5A–F).

Another oxidative stress marker is glutathione (GSH). Increased GSH levels were associated with various types of tumors and were shown to play a role in resistance to chemotherapy [34,35,36]. To determine the effect of NPG on the GSH cycle in PDAC cells, MiaPaca-2 and CD18/HPAF cells were incubated with NPG for 24 h, and both reduced (GSH) and oxidized (GSSG) glutathione levels were measured. NPG treatment reduced the GSH/GSSG ratio, an indication of oxidative stress. In addition, when NPG was combined with HO-1 inhibition, the GSH/GSSG ratio was significantly decreased (*p* < 0.05), indicating a disruption in GSH cycle induced by increased ROS production in both MaiPaca-2 and CD18/HPAF cells (Figure 5G).

### 3.5. Inhibiting HO-1 Augments NPG-Induced Apoptosis in PDAC Cells

**Figure 6:**
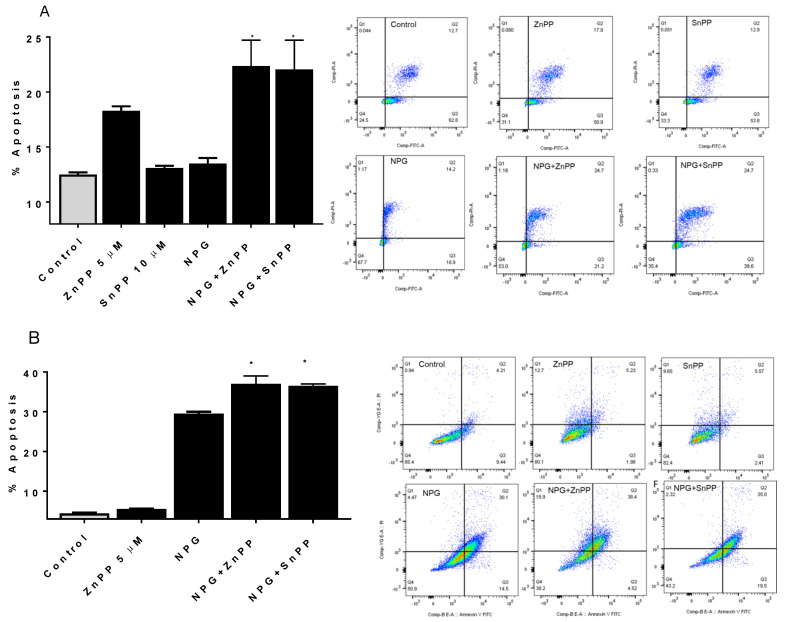

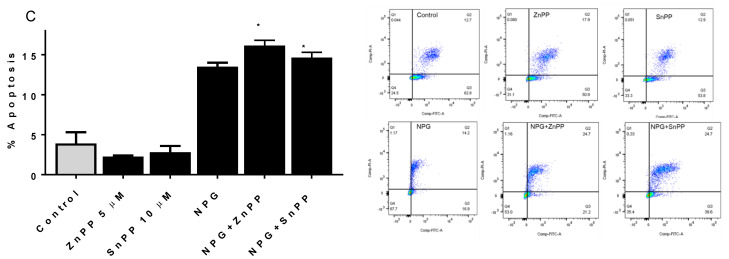
HO-1 inhibition enhances NPG-induced apoptotic cell death. PDAC cells were treated either with NPG, 5 µM ZnPP, or 10 µM SnPP, or a combination for 24 h. Apoptotic cells were detected by flow cytometry using annexin V/PI staining as described in the Materials and Methods. (**A**) CD18/HPAF, (**B**) MiaPaca-2, and (**C**) Capan-1. * *p* < 0.05.

We tested the effects of NPG treatment and HO-1 inhibition on apoptosis in PDAC cells by flow cytometry. Previously, we reported that inhibiting HO-1 induced apoptosis in PDAC cells and enhanced the apoptotic effects of GEM [12]. Figure 6 shows that NPG treatment induced apoptosis of PDAC cells, which was further increased upon addition of HO-1 inhibitor (*p* < 0.05) (Figure 6A–C). In CD18/HPAF, addition of ZnPP to NPG-treated cells increased apoptosis by 60% compared to NPG alone, and SnPP increased apoptosis by 59% in NPG-treated PDAC cells (Figure 6A). Similar results were observed in MiaPaca-2 and Capan-1 cells, although to a lesser extent (*p* < 0.05) (Figure 6B,C). These results indicate that inhibiting HO-1 enhances the apoptotic effect induced by NPG.

### 3.6. HO-1 Inhibition Augments NPG Cytotoxicity in the PDAC Orthotopic Xenograft Mouse Model

**Figure 7:**
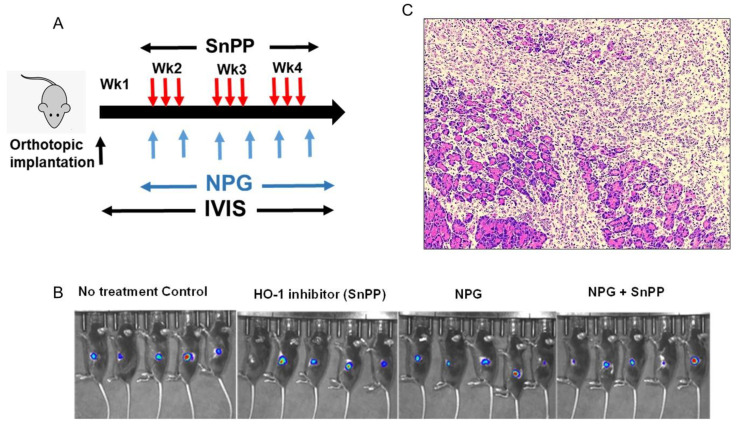

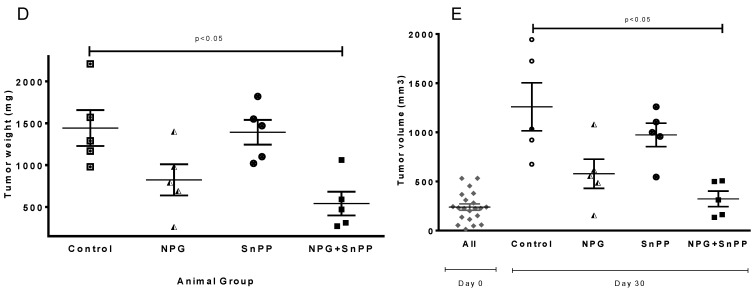
HO-1 inhibition reduces tumor burden in combination with NPG in animal models of PDAC. Luciferase-expressing KPC cells were used to create an in vivo syngeneic mouse model of PDAC. (**A**) Representation scheme of mice treatment. Mice were treated with GEM twice a week at 60 mg/kg/dose, nab-paclitaxel twice a week at 5mg/kg, and SnPP every other day at 5 mg/kg. (**B**) IVIS images showing mice at day 7 after tumor inoculation showing tumors growing in all mice groups. (**C**) Representative H&E staining of in orthotopic primary pancreatic cancer. (**D**) The graph shows tumor weights obtained from mice at the end of experiment (mean ± SEM. *n* = 5 per group, *p* < 0.05. (**E**) The graph shows ex vivo tumor volumes. Each point represents the volume of each tumor and the lines represent the mean value; *p* < 0.05.

To validate our in vitro findings, we investigated the anti-tumor efficacy of HO-1 inhibitors in combination with NPG in a preclinical model of PDAC. Luciferase-expressing KPC mouse cells (KPC-luc) were used for orthotopic implantation of cells into the pancreas of C57BL/6 mice. Mice were treated with NPG twice a week (60 mg/kg/dose of GEM and 5 mg/kg of nab-paclitaxel) and SnPP every other day at 5 mg/kg (Figure 7A). Treatment with NPG, an HO-1 inhibitor (SnPP), or a combination of both agents, and vehicle control began 1 week after implantation and lasted for 4 weeks. Tumor growth was verified using IVIS imaging at day 7 (Figure 7B). No significant weight loss or signs of acute or delayed toxicity were observed in any mouse during treatment. A representative H&E staining of orthotopic primary pancreatic cancer is shown in Figure 7 (Figure 7C).

After 3 weeks, treatment with either NPG or SnPP showed little anti-tumor effect as compared to control group, (mean tumor weight; vehicle control: NPG: SnPP, 1.44 ± 0.21 g: 0.82 ± 0.18 g: 1.39 ± 0.14 g, (Figure 7D). However, mice treated with both NPG and SnPP experienced a significant inhibition of tumor growth (*p* < 0.05) (mean tumor weight, vehicle control: NPG + SnPP, 1.44 ± 0.21 g: 0.54 ± 0.14 g, Figure 7D). Similar results were obtained by measuring tumor volumes as compared to controls (Figure 7E).

### 3.7. Transcriptome and Enrichment Analyses between NPG and NPG with HO-1 Inhibition Treatment Groups

**Figure 8:**
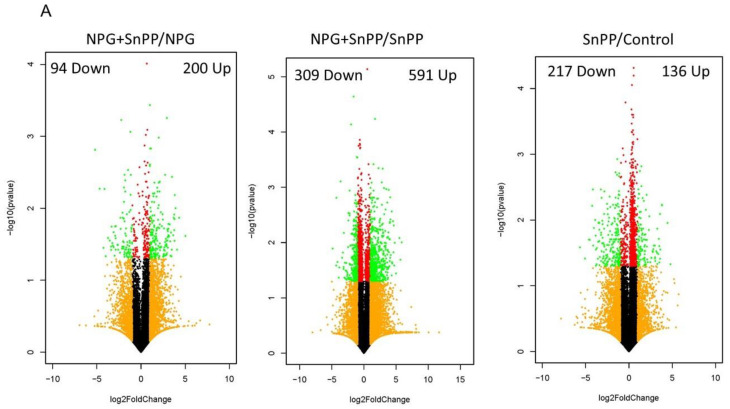

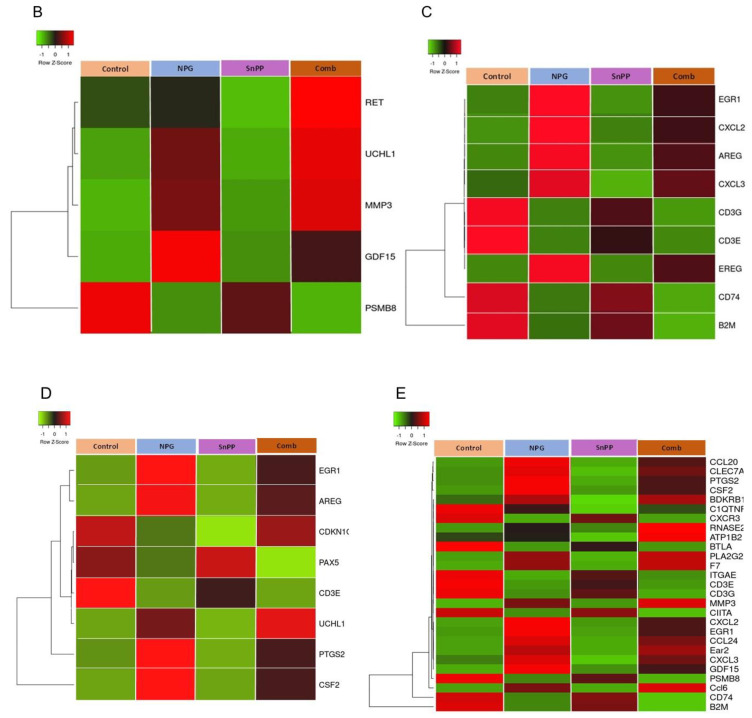
Identification of difference in pancreatic tissue gene expression in different treatment groups. (**A**) Volcano plot of differentially expressed genes in combined treatment (NPG + SnPP) vs. NPG-alone mice. A total of 200 genes showed increased expression and 94 genes showed decreased expression (left). When combined treatment was compared to HO-1 inhibition alone, a total of 591 genes showed increased expression and 309 genes showed decreased expression (middle). Finally, when HO-1 inhibitor-treated mice were compared to non-treated controls, a total of 136 genes showed increased expression and 217 genes showed decreased expression (Right). (**B**–**E**) Heat map showing the effect of NPG, SnPP, and their combination on the clustering of differentially expressed genes in orthotopic pancreas tumors. RNA-Seq was performed on tumors obtained from the non-treated control, NPG, SnPP, and NPG+SnPP (combined)-treated mice. (**B**) Heat maps for differentially expressed genes from for apoptosis. (**C**) Cancer cell invasion and proliferation, (**D**) cell cycle, (**E**) and immune cell trafficking gene pathways are shown for the 4 treatment groups (control, NPG, SnPP, and combined; *n* = 2 each). The color gradient represents low expression (green) to high expression (red).

To explore the potential mechanisms of HO-1 inhibition, RNA sequencing (RNA-seq) was performed in pancreatic tissues obtained from different treatment groups. The bioinformatic analysis of RNA-seq data from mice treated with NPG as compared to NPG with HO-1 inhibitor (SnPP) identified 200 up-regulated genes and 94 down-regulated genes (Figure 8A, left panel). When NPG with SnPP was compared to SnPP alone, 309 down-regulated genes and 591 up-regulated genes were identified (Figure 8A, Middle panel). Mice treated with SnPP alone showed 136 up-regulated genes and 217 down-regulated genes as compared to control non-treated mice (Figure 8A, Right panel) (absolute fold change, >2, up-regulated genes *p* < 0.05 and log2(FC) > 1) and down-regulated genes, *p* < 0.05 and log2(FC) < −1).

The transcriptome of untreated control animals was compared to SnPP, NPG, and NPG+SnPP-treated mice to identify differentially expressed genes using a log2 fold-change cut off of ≤−1 and ≥1. We identified the molecular pathways involved in the antitumor effects of NPG + HO-1 inhibitor. Multiple apoptosis-promoting genes were up-regulated with NPG combined with HO-1 inhibitor (RET, UCHL1, MMP3, and GDF15). Anti-apoptotic genes such as PSMB8 were down-regulated (*p* < 0.05) (Figure 8B).

Combining NPG with HO-1 inhibitor demonstrated down-regulation of genes involved in cancer cell invasion and proliferation (EGR1, CXCL2, AREG, CXCL3, EREG, CD3e, CD3g, CD74, and B2M) as compared to NPG or to control animals (Figure 8C).

Additionally, multiple cell cycle-controlling genes were modulated in HO-1 inhibitor+ NPG combination-treated tumors. These included the up-regulation of cell cycle-inhibitory genes (EGR1, AREG, CDKN1C, UCHL1, PTGS2, and CSF2) and down-regulation of cell cycle-promoting genes (CD3e and PAX5) (Figure 8D).

Interestingly, inhibiting HO-1 in combination with NPG affected multiple immune cell trafficking genes. As shown in Figure 8E, we noticed alterations in expression levels of genes involved in macrophage migration, T cell activation, and chemokine secretion, including: B2M, CD74, PSMB8, CIITA, CD3G, CD3E, ITGAE, BTLA, CXCR3, C1QTNF6, Ccl6, CLEC7A, CXCL3, CCL20, PTGS2, EGR1, Ear2, CXCL2, CCL24, GDF15, CSF2, MMP3, ATP1B2, F7, PLA2G2D, BDKRB1, and RNASE2.

Several genes were down-regulated upon combined treatment as compared to control or NPG treatment alone. One gene was CD74, for which expression was observed in 95% of PDAC cases, and gene expression analysis showed that CD74 overexpression in PDAC is considered a marker of poor prognosis [37,38]. A second gene of interest is integrin alpha E, also known as CD103 (ITGAE). CD103 has been reported to be a marker of tumor-infiltrating Tregs in colon cancer [39], and in murine model of PDAC, high levels of CD103 were expressed on 70% of tumoral Treg cells [40]. Another gene product that plays a role in forming an immunosuppressive T-cell phenotype is dectin-1 (encoded by CLEC7A). Inhibition of the dectin-1–mediated signaling was described as a potential immunotherapy strategy for PDAC patients [41]. B- and T-lymphocyte attenuator (BTLA) is an inhibitory receptor that shares structural and functional similarity with CTLA-4 and PD-1, and is expressed in T-lymphocytes [42,43]. Up-regulation of BTLA is involved in the inhibition of anti-tumor immunity in cancer tissues [44]. Interestingly, high plasma levels of BTLA correlate with poor outcome in PDAC [45].

### 3.8. HO-1 Inhibition with NPG Increases In Vivo Apoptosis, Decreases Macrophage and Treg Recruitment, and Increases CD8 T Cell Infiltration

**Figure 9:**
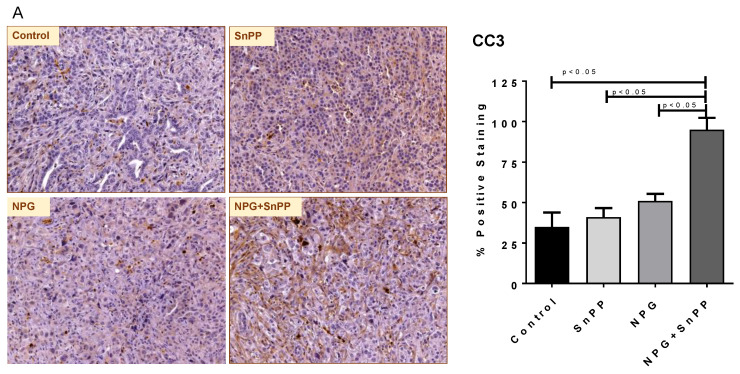

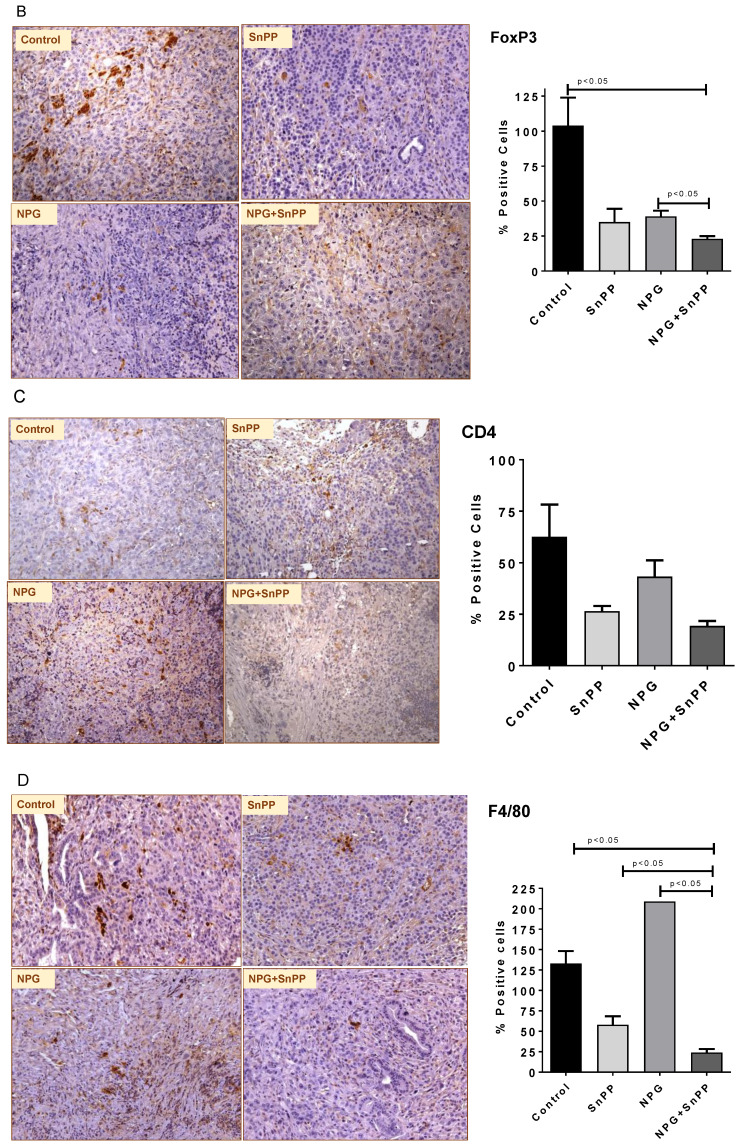

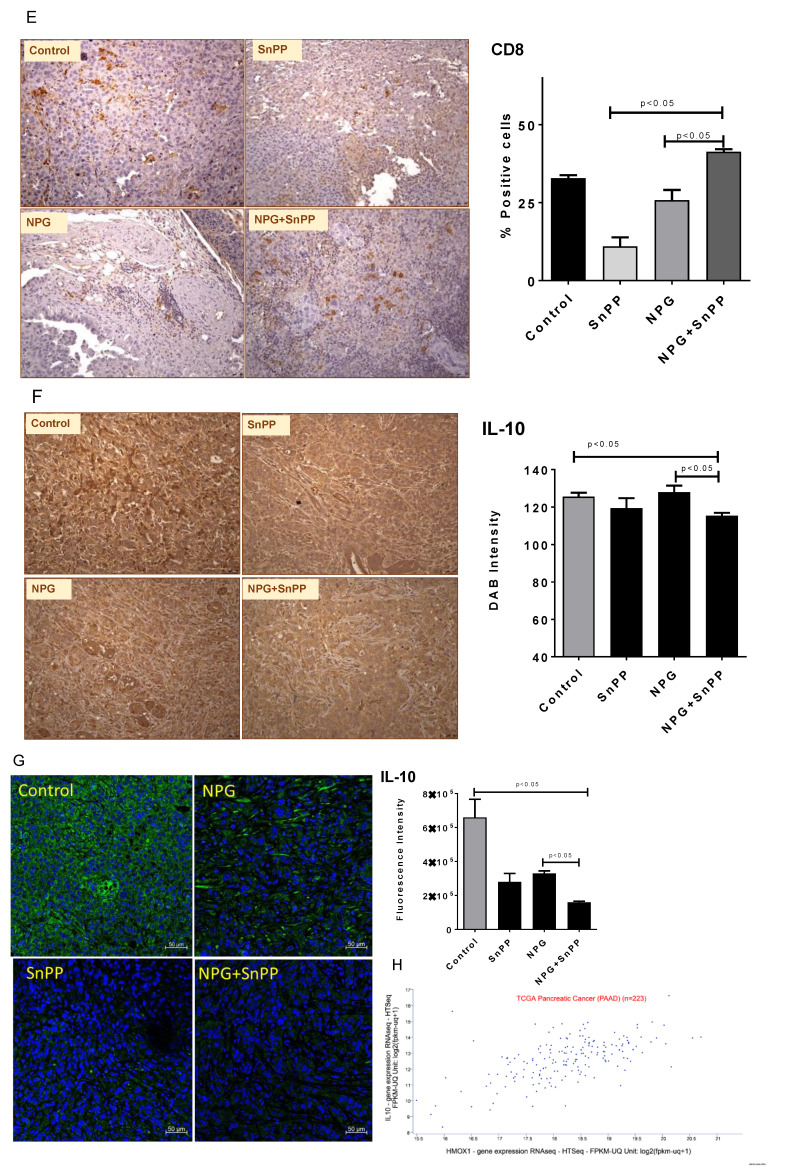
HO-1 inhibition modulates pancreatic TME and regulates immune cell infiltration. (**A**–**G**) IHC images for CC3, FoxP3, CD4, F4/80, CD8, and IL-10. (**G**) IF images for IL-10. The quantification for each staining is shown in the histogram in the right side. Histogram bars represent mean ± SEM. (ANOVA and Tukey’s tests *p* < 0.05, *n* = 5). (**H**) A positive correlation between HMOX1 gene expression and IL-10 levels in PDAC patients (*n* = 223).

To elucidate mechanistic pathways active in the combined treatment of HO-1 inhibition with NPG, we performed IHC analysis to determine apoptosis in tumor tissues using cleaved Caspase-3 (CC3) antibody. Consistent with our in vitro results, the HO-1 inhibitor+NPG combination-treated tumor demonstrated significantly increased apoptosis as compared to the control, SnPP-alone, or NPG-alone mice (*p* < 0.05) (Figure 9A).

A potential role of HO-1 in modulating immune responses has made it an interesting target in several clinical specialties including cancer [46]. Recently it was shown that deletion of HO-1 in the myeloid compartment enhanced the effects of a therapeutic anti-tumor vaccine and increased cytotoxicity in the TME [47]. Our RNA analysis data showed that inhibiting HO-1 had a clear effect on multiple immune-signaling molecules when combined with NPG. To investigate the effect of HO-1 inhibition in combination of NPG on the immune microenvironment of PDAC, we assessed tumor sections from treated and control animals for immune cell infiltration.

It has been shown that PDAC tissues are highly infiltrated by tumor-associated macrophages (TAM) [48,49]. Macrophages play a crucial role in promoting the immunosuppressive TME by contributing to tumor cell resistance to chemo- and radiotherapies [49,50]. Figure 9B shows that the percentage of macrophages (F4/80 positive cells) in formalin-fixed paraffin-embedded pancreatic tumor tissues was significantly reduced in the HO-1 inhibitor and NPG combination treatment group as compared to control and NPG group (*p* < 0.05) (Figure 9B).

Previously, it was reported that differential activities of CD4 + T cells and CD8 + T cells may contribute to tumor immune escape [51,52], and that tumor-infiltrating dendritic cells (DCs) have a tumor-promoting activity through suppressing T-cell function, especially CD8 + T cells [51,52]. Therefore, we sought to profile the tumor-infiltrating CD4 + T cells, CD8 + T cells, and Treg cells.

No significant differences were found in CD4 + T cell populations between different groups (Figure 9C); however, our IHC studies show that combining HO-1 inhibition with NPG significantly reduced the number of infiltrating FoxP3 + cells (Figure 9D), and increased CD8 + T cells as compared to the NPG and SnPP groups (*p* < 0.05) (Figure 9E). This indicates that HO-1 contributes to maintaining the tumor immunosuppressive microenvironment, and inhibiting HO-1 reduces the immunosuppressive effect by increasing CD8 + T cell infiltration and reducing Treg infiltration.

Regulation of immune cell activation is associated with cytokines in the TME. One mechanism by which PDAC may escape immune surveillance is through immune suppression by soluble inhibitory factors such as interleukin (IL-10) and transforming growth factor (TGF)-β [53]. Therefore, we evaluated IL-10 production in PDAC tumors obtained from treated and control mice. Our IHC and IF results show clearly that combining HO-1 inhibition with NPG reduced tissue level of IL-10 (Figure 9F,G). Interestingly, a positive correlation was found between HO-1 expression and IL-10 levels in PDAC patients using TCGA data (Figure 9H).

## 4. Discussion

According to the American Cancer Society, PDAC accounts for about 3% of all cancers in the United States and about 8% of all cancer deaths in 2021 [1]. Moreover, about 47,050 people died of pancreatic cancer in 2020 [54]. Surgery followed by chemotherapy is the current standard of care for resectable PDAC [55]. However, only 20% patients with PDAC are surgically treated at time of diagnosis, due to the fact that most PDAC patients are diagnosed with advanced stage or metastatic disease [4].

Since 2011, a combination of gemcitabine with nab-paclitaxel has been used as a standard regimen for the first-line treatment of metastatic pancreatic cancer [56]. The nanoparticle albumin-bound paclitaxel could disrupt the tumor stromal structure and increase delivery of gemcitabine in PDAC patients [57]. The gemcitabine and nab-paclitaxel combination significantly improves the prognosis of advanced PDAC; however, dose-limiting toxicities and the development of chemo-resistance limit the amount of NPG that can be given to a patient [58]. Therefore, reducing the dose of NPG given to patients by using combination therapy that enhances efficacy would be highly desirable.

One of the fundamental physiological characteristics of tumor cells is the ability to survive under hypoxic conditions [59]. Hypoxia activates a complex network of signaling pathways that includes pathways downstream of HIF-1α [60]. Hypoxia-inducible genes regulate several biological processes including cell proliferation, angiogenesis, apoptosis, and immortalization [8,61]. Expression studies have highlighted HO-1 as one of the critical genes regulated by hypoxia and by HIF-1α [62,63]. HO-1, the inducible isoform of HO, catalyzes the rate-limiting step of heme degradation to biliverdin (converted by biliverdin reductase to bilirubin), CO, and free ferrous iron [64]. Both biliverdin and bilirubin have antioxidant properties. Free iron is sequestered and stored by ferritin, and this prevents production of additional ROS by the Fenton reaction [65,66]. CO has tissue-protective and anti-inflammatory effects [67]. Previously, our lab has shown that hypoxia increased HO-1 expression in different PDAC cells [12], and inhibiting HO-1 enhanced the toxicity of GEM in vitro and in vivo. In this work, we extend our studies to test the effect of HO-1 inhibition in combination with NPG on PDAC cells and the TME.

Our results show that NPG treatment increased HO-1 expression in PDAC cells through the activation of MAPK-p38 signaling pathway. Our study showed that NPG induces HO-1 nuclear translocation. Although this has been reported before in other cancers, we are the first to show this in pancreatic cancer cell lines. The exact role of HO-1 in nuclei remains to be investigated in PDAC. However, others have reported that HO-1 nuclear translocation confers resistance to chemotherapy and induces genetic instability in other cancer cells [68,69].

Our study demonstrated that inhibiting HO-1 in combination with NPG showed increased cytotoxicity to PDAC cells and tumors. We used pharmacological inhibitors for HO-1 (ZnPP, and SnPP) and evaluated the growth behavior of different PDAC cell lines. Our results revealed a significant suppression effect of HO-1 inhibition on PDAC cell survival, indicating a crucial role of HO-1 in tumor growth.

GEM alone or in combination with nab-paclitaxel chemotherapy is used in the clinic as a standard treatment for some patients with PDAC. However, development of tumor resistance to GEM critically limits the efficacy of chemotherapy and leads to disappointing outcomes in PDAC patients [70]. To investigate the role of HO-1 in GEM resistance, we extended our study to include GEM-resistant PDAC cells. Again, inhibiting HO-1 increased the sensitivity of GEM-resistant PDAC cells to NPG. Interestingly, when we studied HO-1 expression in these cells, GEM-resistant cells expressed higher levels of HO-1 as compared to parent cells.

The effect of HO-1 inhibition in modulating PDAC cell survival under treatment with NPG confirms the importance of HO-1 in cancer cell survival [12,71]. Further confirmation comes from our studies showing that increasing HO-1 expression levels by the HO-1 inducer (CoPP) increased PDAC cell survival. Thus, the expression of HO-1 is crucial for PDAC cell survival, and inhibiting HO-1 suppresses PDAC cell survival. Similarly, in our mouse model, combining HO-1 inhibition with NPG reduced tumor growth.

HO-1 was significantly induced when PDAC cells were treated with NPG, indicating that this chemotherapy increases oxidative stress in PDAC cells and as a result induces stress-related enzymes including HO-1. The cytoprotective role of HO-1 in tumor cells against oxidative stress induced by chemotherapeutic agents helps these cells to avoid apoptosis and promotes cell proliferation and metastasis [72,73].

Mechanistically, combining HO-1 inhibition with NPG resulted in higher accumulation of ROS and increased cellular GSSG, indicating a disruption in oxidative stress status. In preclinical mouse models, our laboratory and others have demonstrated that administration of HO-1 pharmacologic inhibitors has antitumor effects and enhances response to chemotherapy [12,74]. The observed effects were due increased ROS, disruptions in the GSH system, increased in vitro and in vivo apoptosis, and decreased expression of stemness markers [12].

GSH is one of the major systems involved in the maintenance of the intracellular redox balance, and plays an important role in cancer progression [75]. Elevated levels of GSH protect tumors by conferring resistance to chemotherapeutic drugs [75,76]. Our results show that the toxic effects of NPG were significantly increased when combined with an HO-1 inhibitor (SnPP) in different PDAC cell lines. We believe that the combination of HO-1 inhibition and NPG caused an increase in steady state levels of ROS and this increase exceeded the metabolic capabilities of the glutathione system to maintain glutathione in the reduced form. This shows the important role of GSH in sustaining cell proliferation and suggests that GSH-disruption through HO-1 inhibition will improve the effectivity of standard chemotherapy in PDAC.

To further support this idea, the thiol antioxidant NAC was able to inhibit the cytotoxicity induced by HO-1 inhibition and NPG. Because NAC is a precursor for GSH synthesis [77], NAC may function by increasing intracellular thiol pools necessary for counteracting the effects of HO-1 inhibition and NPG. Taken together, the data presented in this paper suggest that the cytotoxic effects of HO-1 inhibition in combination with NPG were mediated in part by disruptions in thiol metabolism consistent with oxidative stress, which was reversed by the thiol antioxidant NAC.

The anti-apoptotic action of HO-1 is mediated by multiple mechanisms including decreased intracellular ROS and elevated CO production [78]. Our apoptosis results by flow cytometry analysis showed augmented effects of HO-1 inhibition and NPG treatment as compared to single treatment. Similar results were seen in pancreatic tumor tissues obtained from combined treatment mice and stained by CC3.

The anti-tumor effects of NPG were significantly higher when combined with the HO-1 inhibitor (SnPP) in vivo. Both tumor weights and tumor volumes were decreased as compared to controls. Ex-vivo studies show that inhibiting HO-1 increased apoptosis in pancreatic tumors as compared to control and to NPG alone.

The role of increased HO-1 in modulating the immune system has been described in multiple cancers and is usually associated with poor prognosis [79,80]. Recently, the role of HO-1 in tumor-associated macrophages (TAM) was studied, and it was shown that deletion of HO-1 in the myeloid compartment modulates the immune response and reduces the immunosuppressive TME [47,81].

To better understand the role of HO-1 in modulating the immune system and to overcome the shortcomings of the nude mouse models of PDAC, we used an immunocompetent orthotopic mouse model of PDAC in which luciferase-expressing pancreatic tumor cells from KPC mice were implanted into the pancreas of immunocompetent syngeneic C57BL/6J mice.

Our results demonstrate that combining HO-1 inhibition with NPG significantly decreased tumor-infiltrated macrophages as compared to controls and to NPG alone. This is a very significant result given that PDAC tissues are highly infiltrated by TAMs, which play a role in tumor progression and the tumor-promoting microenvironment [82,83]. This is mainly due to the fact that the majority of TAMs are of the M2 phenotype, which are shown to promote invasion, metastasis, and resistance to chemotherapy [82,83,84]. In general, infiltrating macrophages express inhibitory receptors such as programmed cell death ligand 1 (PD-L1), and therefore may interfere with immunotherapy by inhibiting CD8 + T cells [50]. Additionally, the release of immunosuppressive cytokines such as IL-10 by M2 macrophages promotes Th2 immune responses [50,85]. Activated M2 TAMs have been shown to promote epithelial–mesenchymal transition (EMT) in pancreatic cancer, which contributes to the progression of primary tumors to a metastatic state [86]. Other studies have shown that TAMs play an important role in tumor progression by contributing to tumor resistance to chemotherapies, and as such, targeting TAMs can effectively overcome this resistance [87].

The unique immunosuppressive TME of PDAC attracts immunosuppressive immune cells such as Tregs and MDSCs in the pre-invasive, invasive, and metastatic stages of PDAC [88,89]. Circulating peripheral Treg levels were higher in patients with PDAC as compared to healthy donors [90,91]. Our results show clearly that combining HO-1 inhibition with the standard chemotherapy reduced tumor-infiltrated Tregs. This indicates that HO-1 plays an important role in inducing the immunosuppressive TME. Although the exact mechanism of how HO-1 increases Treg and FoxP3 expression is still not well understood, a relationship between them has been described previously [92,93].

Cytotoxic CD8 + T-cells are important components in recognizing and attacking tumor cells [94]. It is known that Tregs promote PDAC development by suppressing anti-tumor immunity of CD8+ T cells [95]. In xenograft murine models, the presence of CD8 + T cells was associated with reduced tumor growth [96]. Previous studies in PDAC showed that anti-tumor activity of CD8 + T cells favors better clinical outcome and patient survival [51,95,97]. Our results show that inhibiting HO-1 in combination with NPG increased tumor infiltrated CD8 + T cells. This could in part explain the smaller tumor size in this group. These data collectively show that HO-1 inhibition increases CD8 + T cell infiltration in PDAC tissues and acts as a potential modulator of immune responses in combination with NPG.

Since cytokines are responsible for the induction of immune cells against tumors, we evaluated the levels of IL-10 in tumor sections of experimental and control animals. High levels of serum-immunosuppressive cytokines such as IL-10 were reported to play a key role in PDAC development [53,98]. IL-10 induces an immunosuppressive microenvironment in PDAC patients, reduces effector cell function and shifts toward Th2 cytokines, and therefore helps tumor cells to escape immune recognition [53,99]. Multiple studies have shown an association between high expression of IL-10 and the ability of PDAC to escape immune recognition [100]. In clinical studies, the immunosuppressive function of IL-10 in tissue and serum of PDAC patients possesses a clear positive correlation with tumor stage and poor differentiation status [100,101]. In addition, IL-10 secreted by Treg cells reduces T cell expansion by suppressing IL-2 production in T cells and induces T cell anergy [102]. The interaction between HO-1 and IL-10 has been reported. HO-1 induction prompts macrophage polarization into an IL-10-producing anti-inflammatory (M2) phenotype [103]. In murine macrophages, treatment with IL-10 induces HO-1 in a dose-dependent manner through the activation of MAPK p38 activity [104]. However, the relationship between IL-10 and HO-1 in pancreatic cancer has not been clearly identified. Our results show that inhibiting HO-1 in combination with standard chemotherapy reduces PDAC IL-10 levels and therefore modulates the TME.

## 5. Conclusions

**Figure 10:**
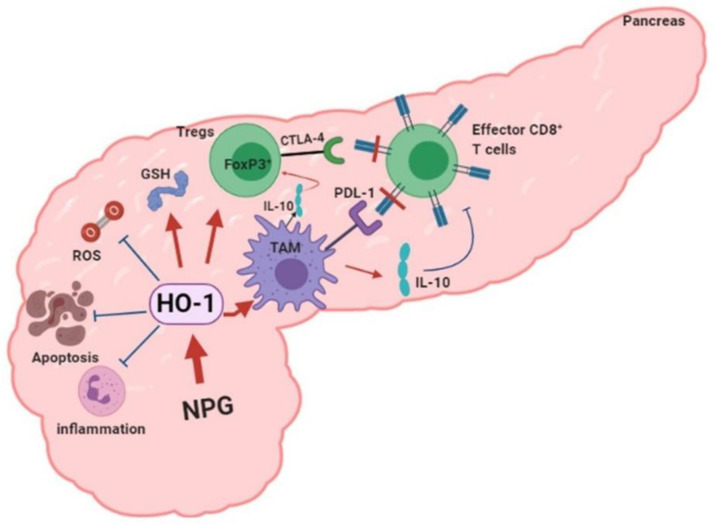
Proposed model of HO-1 in PDAC. Chemotherapy: NPG induces HO-1 in PDAC tissue. HO-1 provides the antioxidant environment that scavenges ROS, inhibits inflammation, and reduces the apoptosis which favors tumor growth. Induced HO-1 also increases TAM infiltration, FoxP3 expression, and IL-10 production, creating an immunosuppressive TME. Inhibiting HO-1 reverses these effects and reduces the immunosuppressive TME. (Figure was created using BioRender.com).

In conclusion, our data provide new mechanistic insights into the impact of HO-1 inhibition on tumor progression, drug resistance, and the TME in PDAC. We propose a model in which HO-1 induction by NPG reduces oxidative TME by scavenging ROS, decreases apoptosis, and increases tumor-infiltrated Tregs and macrophages. Inhibiting HO-1 would increase tumor-killing and reduce IL-10 production directly or indirectly by reducing TAMs (Figure 10).

## Data Availability

The data presented in this study are available in this article.

## Supplementary Materials

The following are available online at https://www.mdpi.com/article/10.3390/cancers13092264/s1, supplementary file 1: The western bolt of Figures 2 and 4.
