# Supplementary figures and images for: Heme Oxygenase-1 Inhibition Potentiates the Effects of Nab-Paclitaxel-Gemcitabine and Modulates the Tumor Microenvironment in Pancreatic Ductal Adenocarcinoma
Source: Cancers (Basel). 2021 May 8;13(9):2264. doi: 10.3390/cancers13092264 (PMC8125955; doi:10.3390/cancers13092264)

Figure 2 D

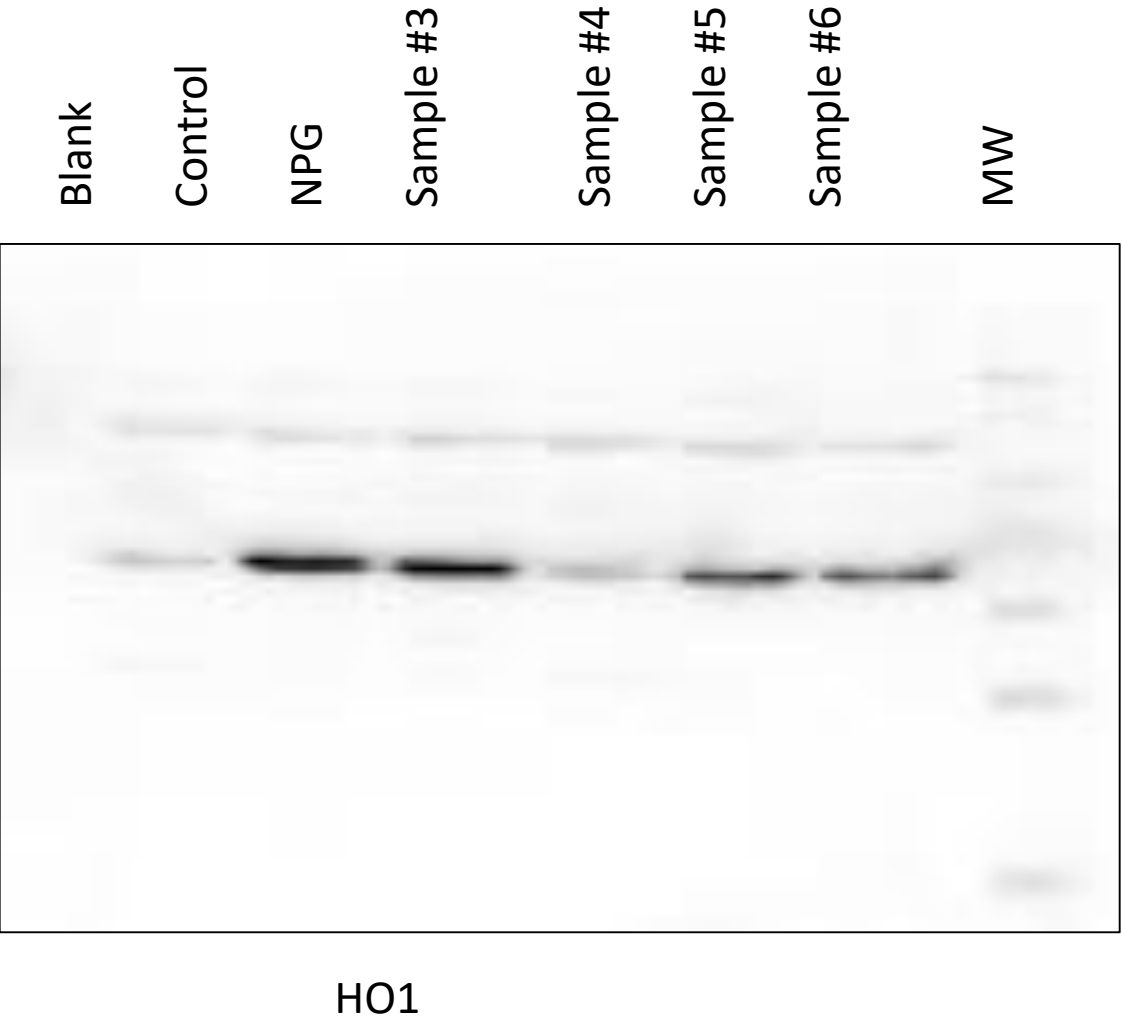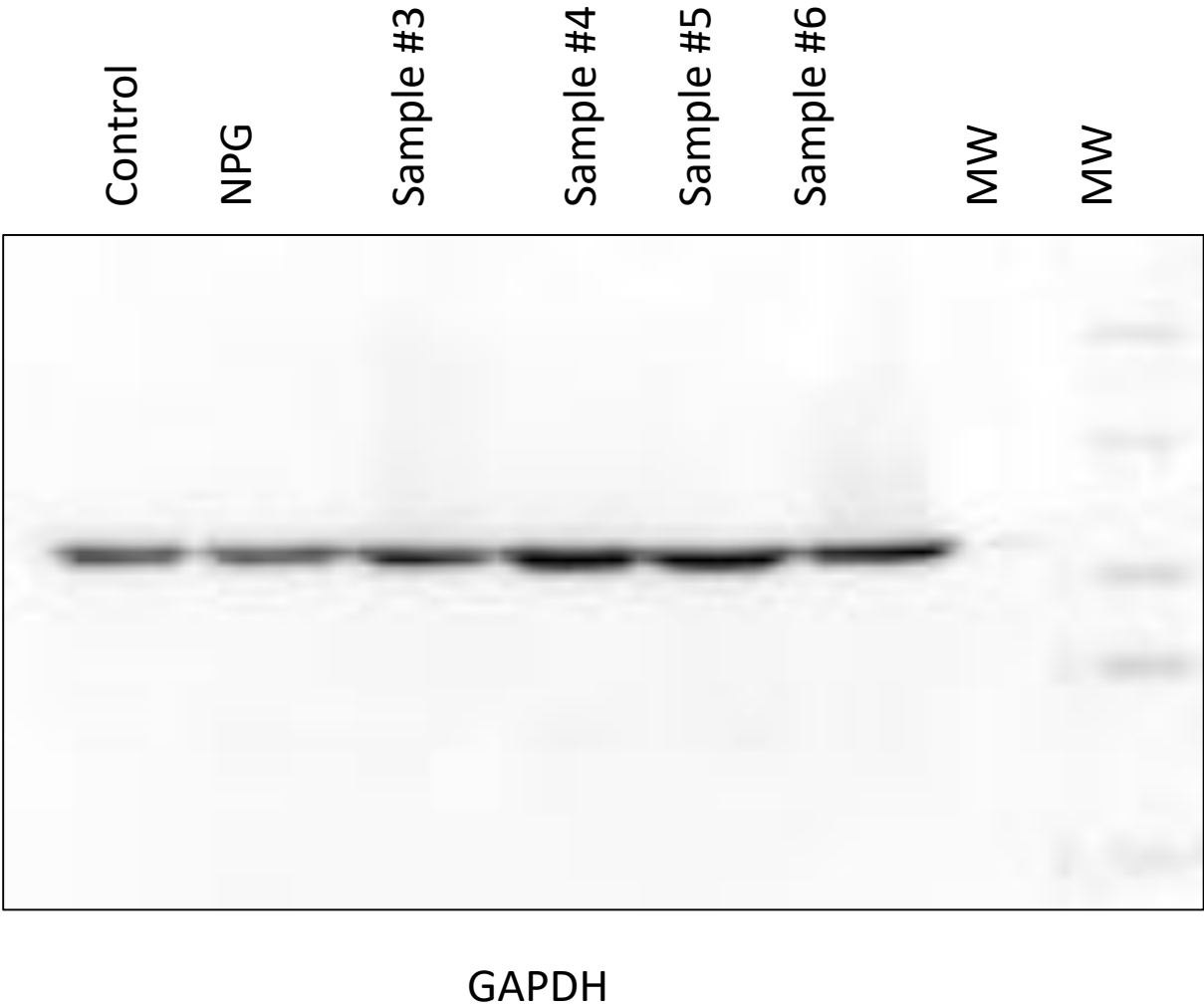

Figure 2 E

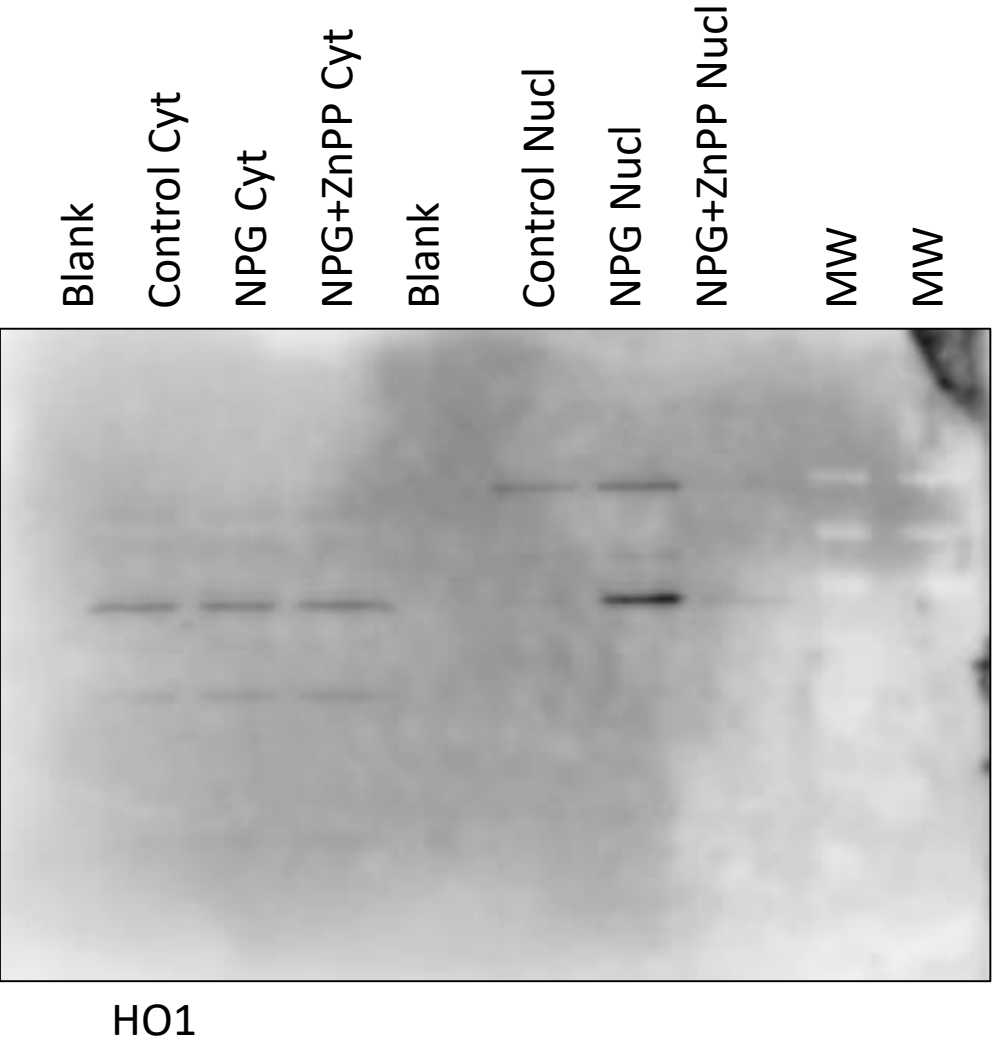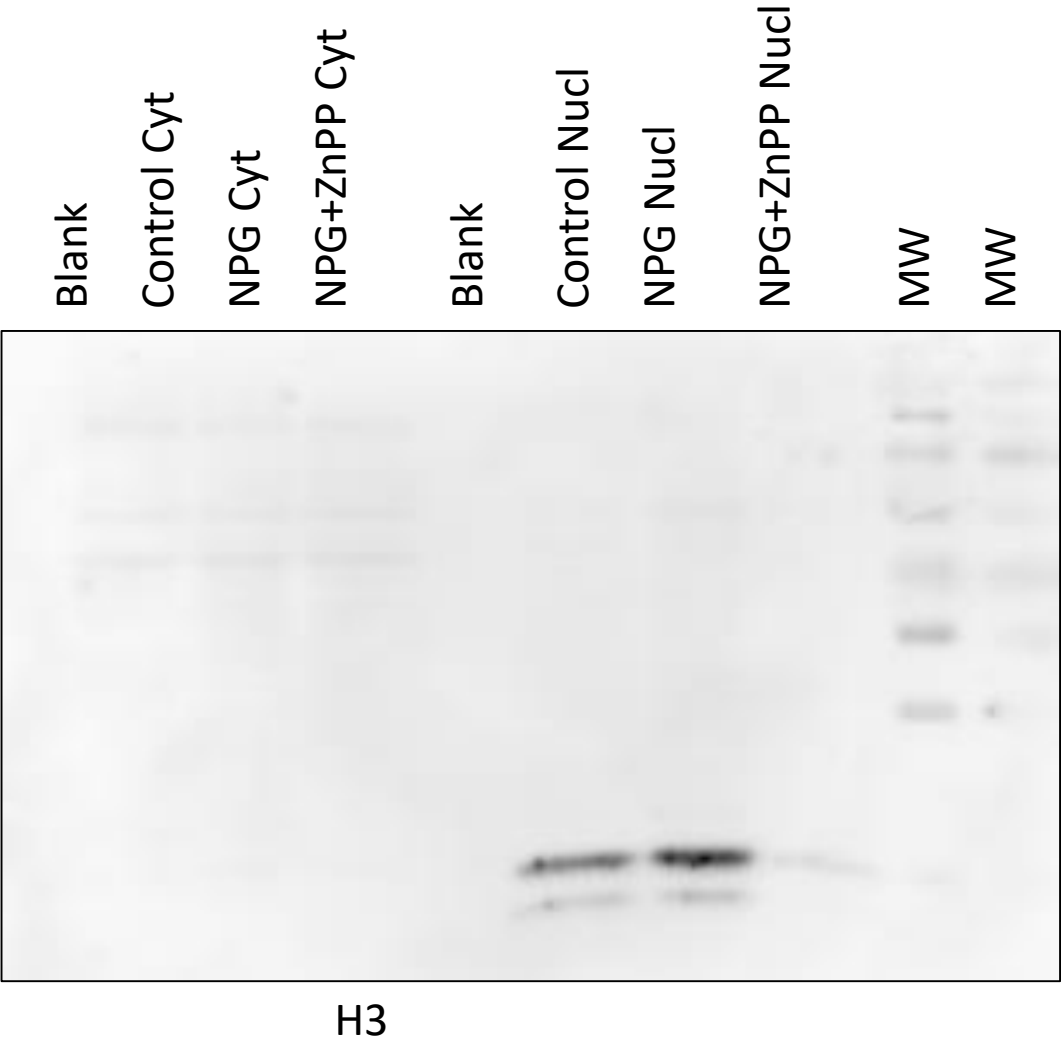

Figure 2 E

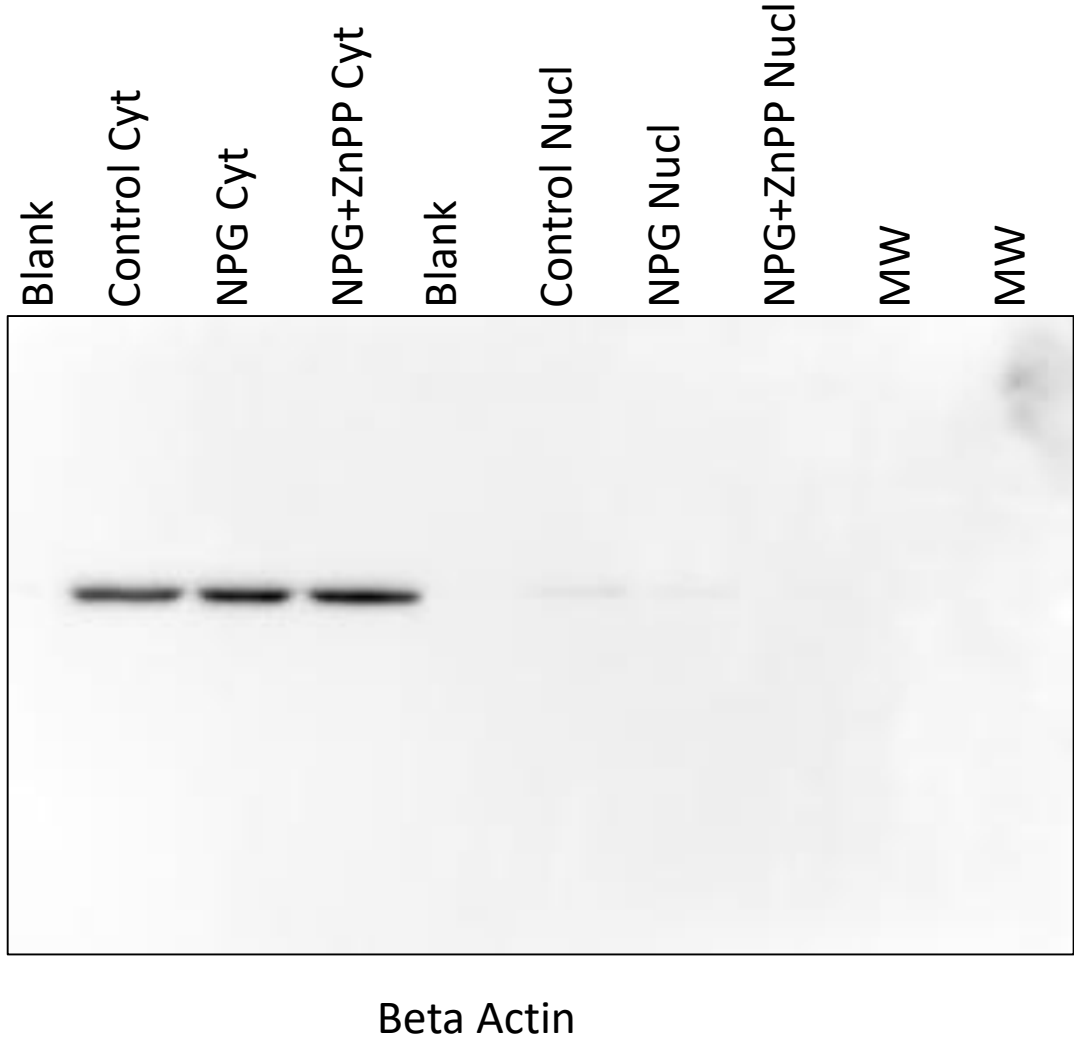

Figure 2 F

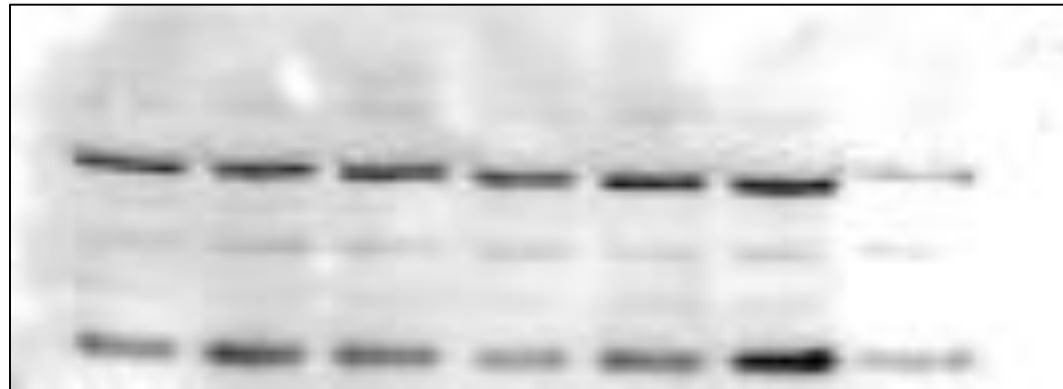

HO1, selected top half

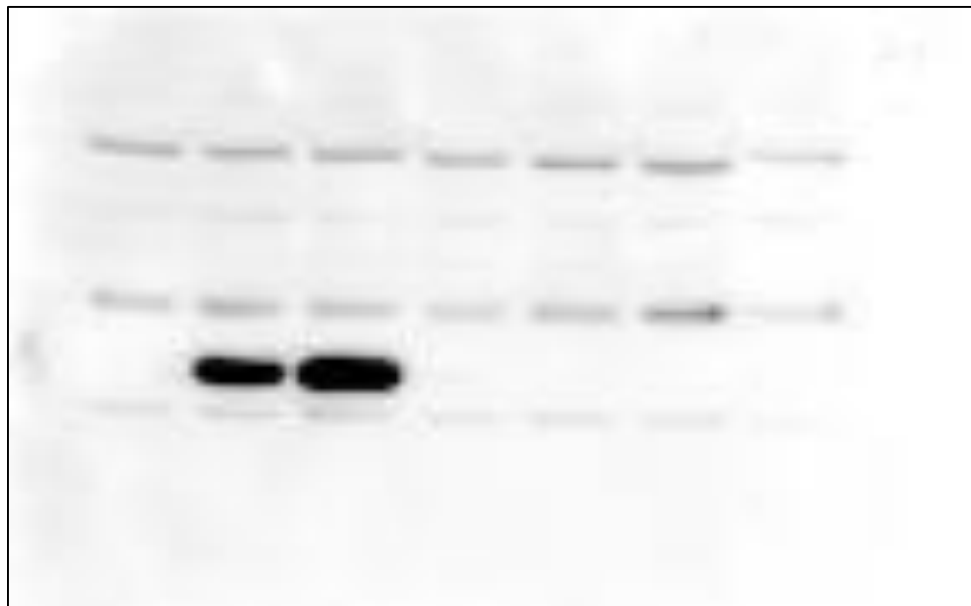

HO1

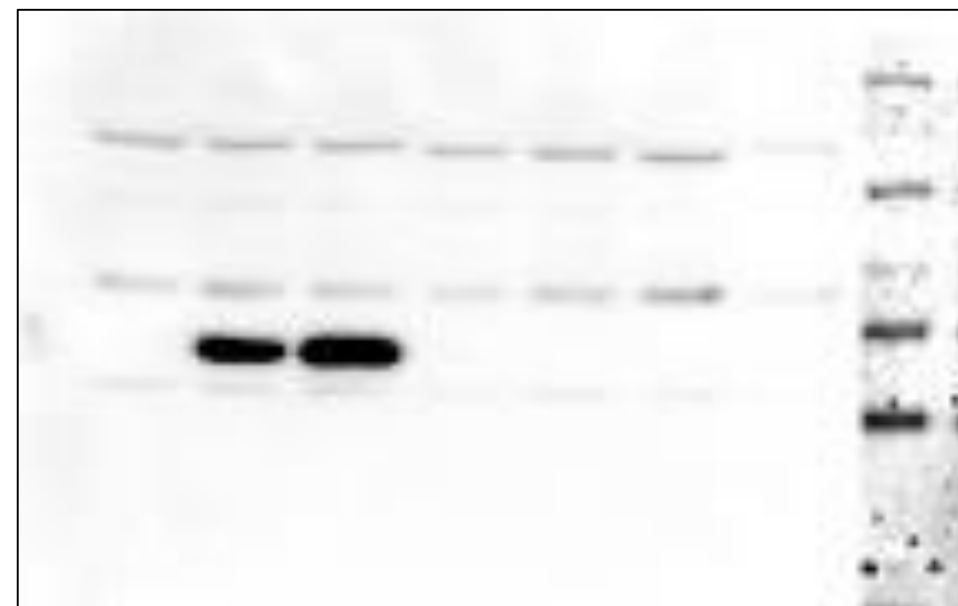

HO1 full-Mwt

Figure 2 F

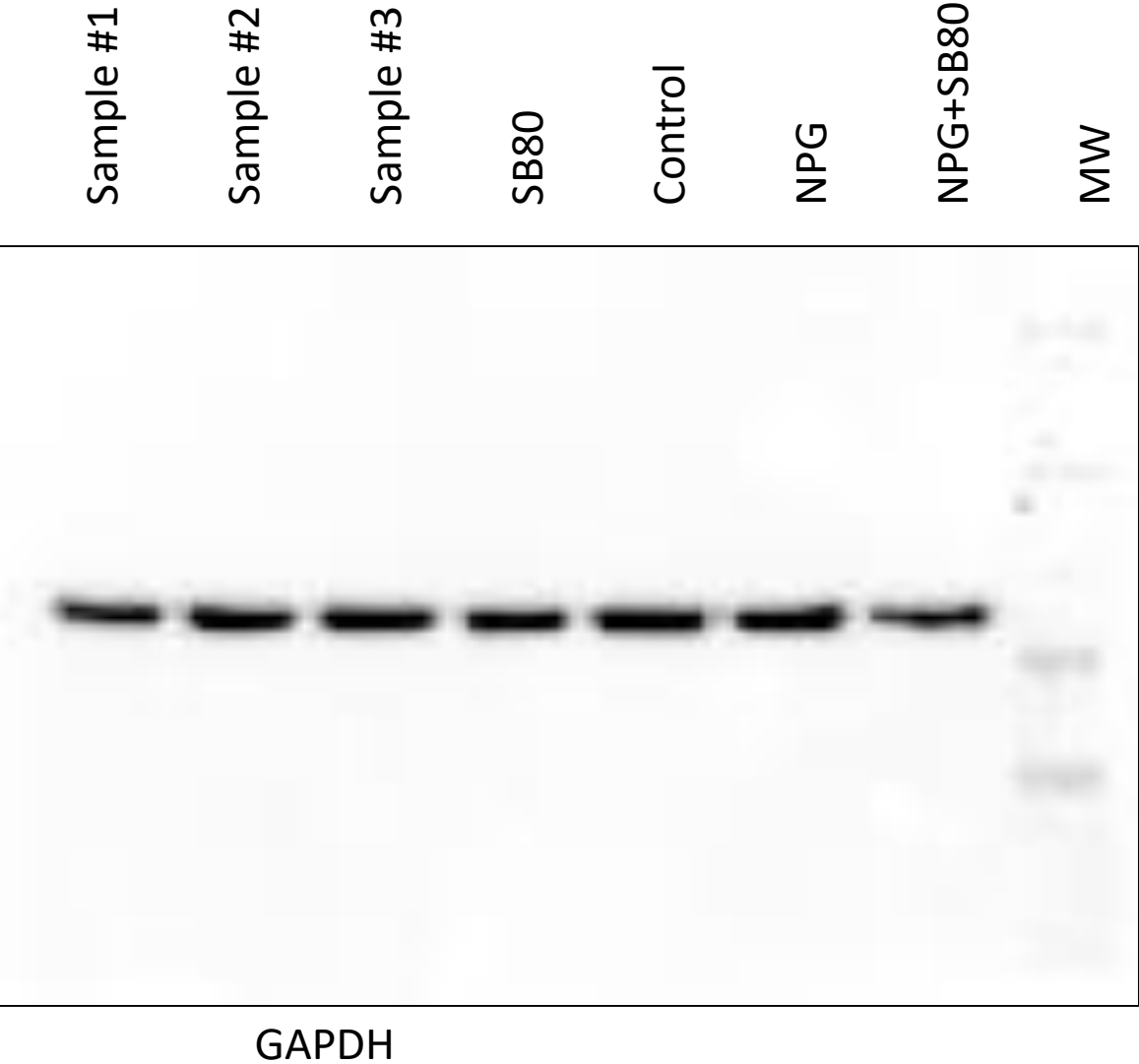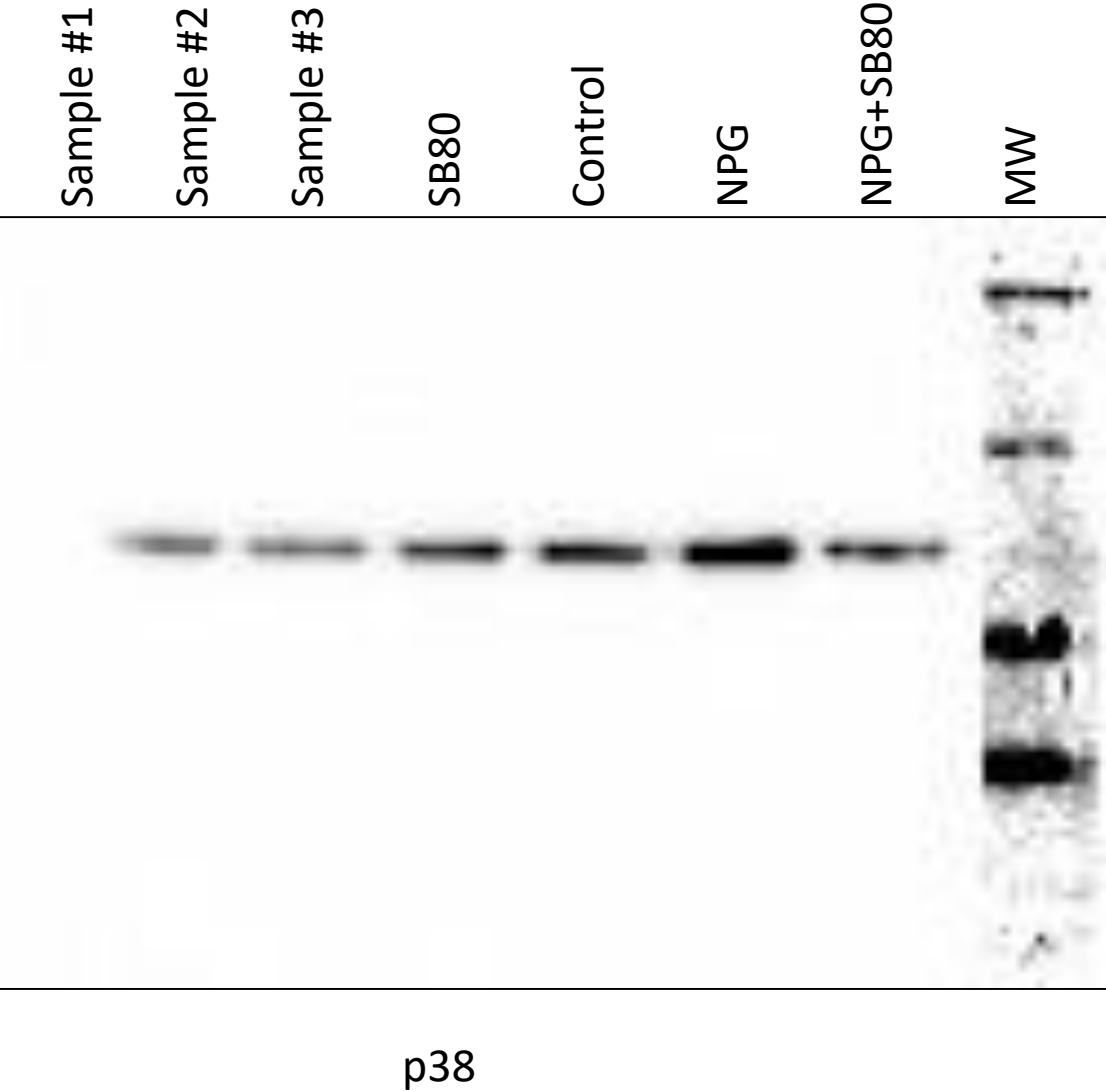

Figure 4

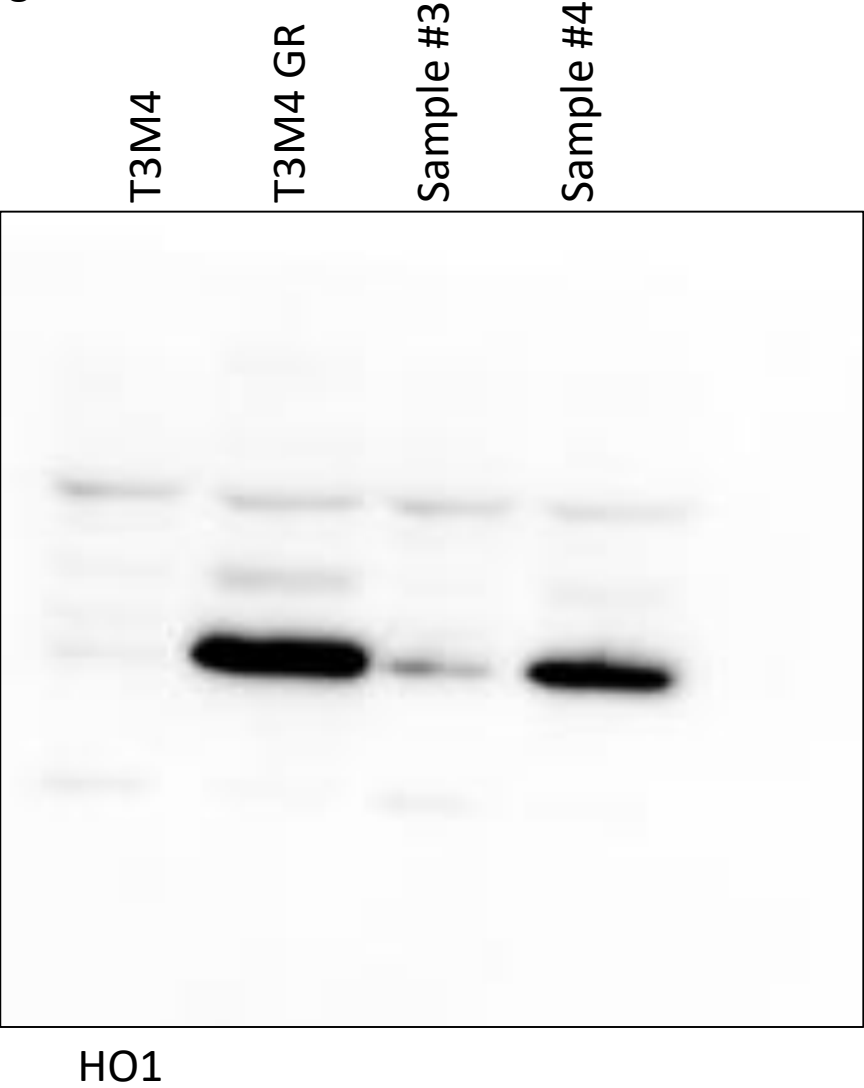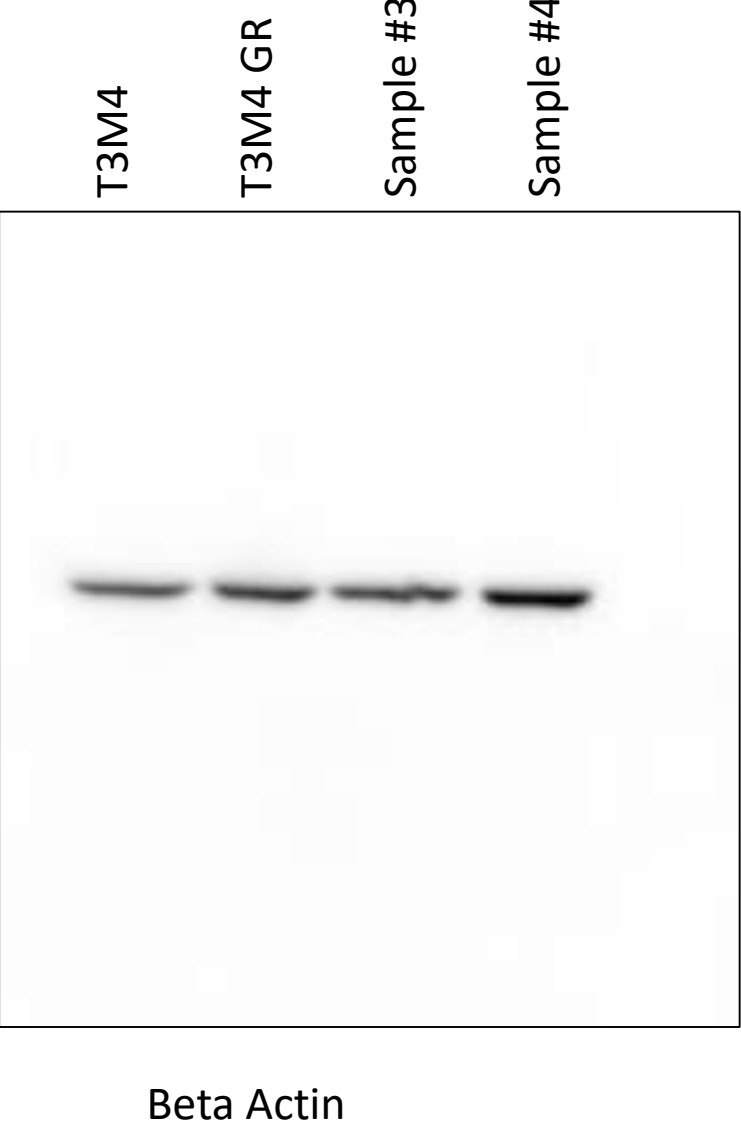

Supplement: Supplementary file 1 [file cancers-13-02264-s001.zip › cancers-1173582-supplementary.pdf]
